# Pangenome and genome variation analyses of pigs unveil genomic facets for their adaptation and agronomic characteristics

**DOI:** 10.1002/imt2.257

**Published:** 2024-12-26

**Authors:** Dong Li, Yulong Wang, Tiantian Yuan, Minghao Cao, Yulin He, Lin Zhang, Xiang Li, Yifan Jiang, Ke Li, Jingchun Sun, Guangquan Lv, Guosheng Su, Qishan Wang, Yuchun Pan, Xinjian Li, Yu Jiang, Gongshe Yang, Martien A. M. Groenen, Martijn F. L. Derks, Rongrong Ding, Xiangdong Ding, Taiyong Yu

**Affiliations:** ^1^ Key Laboratory of Animal Genetics, Breeding and Reproduction of Shaanxi Province, Laboratory of Animal Fat Deposition & Muscle Development, College of Animal Science and Technology Northwest A&F University Yangling Shaanxi China; ^2^ State Key Laboratory of Animal Biotech Breeding National Engineering Laboratory for Animal Breeding, College of Animal Science and Technology China Agricultural University Beijing China; ^3^ Key Laboratory of Vertebrate Evolution and Human Origins Chinese Academy of Sciences Beijing China; ^4^ Institute of Subtropical Agriculture Chinese Academy of Sciences Changsha Hunan China; ^5^ Centre for Quantitative Genetics and Genomics Aarhus University Aarhus Denmark; ^6^ Department of Animal Science, College of Animal Science Zhejiang University Hangzhou China; ^7^ Sanya Institute Hainan Academy of Agricultural Science Sanya Hainan China; ^8^ Animal Breeding and Genomics Wageningen University and Research Wageningen The Netherlands

## Abstract

The development of a comprehensive pig graph pangenome assembly encompassing 27 genomes represents the most extensive collection of pig genomic data to date. Analysis of this pangenome reveals the critical role of structural variations in driving adaptation and defining breed‐specific traits. Notably, the study identifies *BTF3* as a key candidate gene governing intramuscular fat deposition and meat quality in pigs. These findings underscore the power of pangenome approaches in uncovering novel genomic features underlying economically important agricultural traits. Collectively, these results demonstrate the value of leveraging large‐scale, multi‐genome analyses for advancing our understanding of livestock genomes and accelerating genetic improvement.

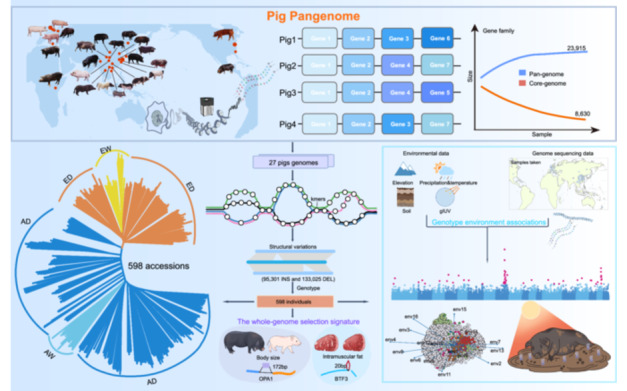

## ETHICS STATEMENT

The ethics application (XN2023‐0101) was approved by the Animal Ethical and Welfare Committee of Northwest A&F University.

To the Editor,

The pig is one of the most important domestic animals [1]. Pig breeds show distinct advantageous traits, including adaptation to high‐altitude environments [2], tolerance to extreme temperatures [3], and excellent meat quality [4]. This genetic diversity provides significant potential for improvement in breeding. The pig reference genome (a Duroc pig) has undergone continuous improvements since its initial release [5], which provided critical insights into the genetic traits. However, the genetic divergence between Asian and European pig breeds restricts us from comprehensively studying genomic variation across diverse pig populations. Although short‐read sequencing has accelerated the discovery of genetic variants, it introduces an inherent bias: the characterized variants are disproportionately skewed toward single‐nucleotide polymorphisms (SNPs) and small insertions/deletions (Indels) [6]. Studies have shown that structural variations (SVs) play crucial roles in the agronomical traits of plants and the economic traits of domestic animals [7]. Recently, pangenome analysis has evolved from detecting genomic variations to an efficient method for SV genotyping, enabling comprehensive identification of SVs and aiding the development of new breeding varieties [8].

Pig pangenome studies have the potential to answer this scientific question, but their limitations in terms of sample size and genome quality have hindered comprehensive analyses [9]. To address this, we constructed a pangenome (27 genomes) incorporating four newly assembled pig genomes and we identified 295.97 Mb of novel sequences and 276,032 nonredundant SVs. The analysis of environmental association showed that SVs have a stronger correlation compared with SNPs and Indels, and SV analysis identified two soil silt‐associated genomic hotspots. Additionally, *BTF3*, a candidate gene that impacts intramuscular fat (IMF) deposition, was revealed through SV and gene function analyses. Our study demonstrated pangenome and SVs as crucial determinants of characteristics, which provided a valuable data resource for domestic animals' genomic research.

## RESULTS AND DISCUSSION

### Genome assembly and annotations

To address the gap in pig genetic resources, we generated genome assemblies for three Asian pig breeds (Bamei (BM), Juema (JM), and Hanjiang Black (HJB)), and one commercial breed (Large White (LW)). The contig N50 of the four genomes ranges from 48.2 to 81.2 Mb, and the benchmarking universal single‐copy orthologs (BUSCO) are 97.8 − 98.0%, which are comparable to the quality of the pig reference genome (*Sscrofa*11.1) (96.9%) (Table S1). The genomic collinearity analysis showed that these genomes have high consistency with *Sscrofa*11.1 (Figure S1A). We further ordered and arranged the contigs of HJB, LW, and 10 genomes from our previous study (BUSCO: 95.5−97.7%) into chromosomes based on their synteny to the reference genome (Figure S1C, Table S1). Although the 14 new genomes contained more gaps (115−668) in autosomal regions than Sscrofa11.1 (108 gaps), their contig N50 values (20.19−81.24 Mb) were comparable to the reference genome (48.23 Mb) (Table S1), and the repeat content in these pig genomes (41.83%−42.73%) was comparable to *Sscrofa*11.1 (41.87%). The total repeat length varied substantially (860.29−1217.99 Mb) (Table S2), but the length of non‐repeat sequences was similar (1469.46−1676.22 Mb) (Figure S1B). We annotated 25,586 and 25,406 protein‐coding genes in the BM and JM pig genomes, respectively; a pipeline was used to annotate other genomes without annotation. BUSCO analysis showed that our annotations achieved an average completeness of 94.49% (Table S3). The genomes contained 1023–2260 noncoding RNAs (ncRNAs), predominantly small nuclear RNAs (snRNAs) (42.46%–57.30%) (Table S4). These high‐quality genome assemblies and annotations present a robust resource in future genomic studies.

### Pig graph pangenome construction and analysis

**FIGURE 1:**
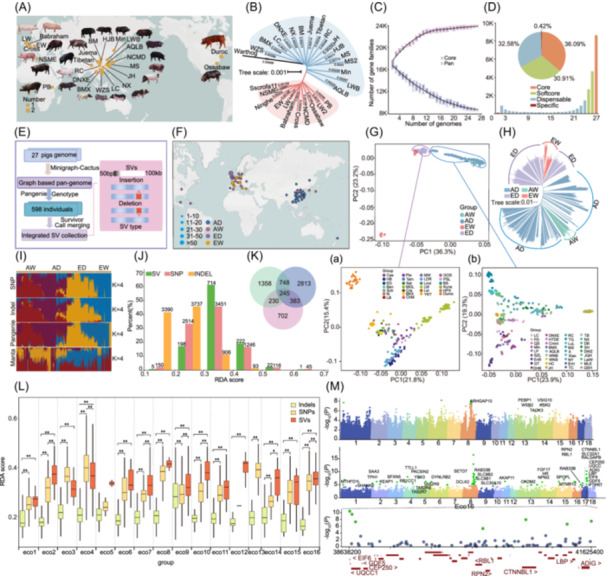
Construction of the pig pangenome and structural variation (SV) analysis. (A) Geographic distribution of 27 pig genomes. (B) Mash‐based phylogenetic tree derived from 27 pig assemblies. (C) Simulated increase in the pangenome size and decrease in core‐genome size. (D) Composition of the pig pan‐genome. (E) SV genotyping pipeline for the pangenome. (F) Geographic distribution of 598 pig accessions in SV analysis. AW, Asian wild pigs; AD, Asian domestic pigs; ED, European pigs; EW, European wild pigs. (G) Principal component analysis (PCA) of 598 pig accessions, with the results for European and Asian pig populations shown in (a) and (b), respectively. The group details are provided in Table S23. (H) Neighbor‐joining (NJ) phylogenetic tree of 598 pig accessions. (I) Admixture analysis of 598 pig accessions (*K* = 4) using single‐nucleotide polymorphisms (SNPs), insertions/deletions (Indels), and SVs sets detected by Pangenie and Manta. (J) Stacked bar plot showing the redundancy analysis (RDA) percentage of associated SNPs, Indels, and SVs. The *x*‐axis shows the RDA scores of SNPs, SVs, and Indels, reflecting their association with environmental variables. (K) Venn diagram showing overlaps of candidate genes among associated SNPs, Indels, and SVs. (L) Box plots of RDA results for associated SNPs, Indels, and SVs across Eco1–Eco16 variables. Statistical significance was evaluated through post‐hoc analysis using the Bonferroni correction for multiple comparisons. The y‐axis shows the RDA scores of SNPs, SVs, and Indels, reflecting their association with environmental variables. Adjusted *p*‐values were considered significant at *p < 0.05 and **p < 0.01. (M). Plots of log_10_ (*p*) from genome‐wide association mapping between SVs and Eco16 variables: the top shows SNPs versus Eco16 variable, the middle shows SVs versus Eco16 variable, and the bottom shows a closer look at the hotspot region on chromosome 17.

To better represent diverse pig breeds, including BM, JM, HJB, and LW, we constructed a pangenome using 27 assemblies from Asian (17), European (9), and African (1) pigs (Figure 1A). Mash analysis showed higher genetic diversity in Asian breeds than in European breeds. Some Asian breeds (Min, Anqingliubai, and Laiwu) showing a closer relationship to European pigs; the largest genetic distance was between Wuzhishan and Duroc (0.005826) (Figure 1B). The Minigraph–Cactus (MC) pangenome has 178,175,328 nodes and 253,272,873 edges, with 295.97 Mb of a non‐reference sequence (Table S5). MC generated the longest non‐repeat sequence (133.11 Mb) with the lowest repeat ratio (55.03%) (Figure S1E, Table S6). BM contributed the highest additional sequence (189.1 Mb), while HJB contributed as little as 9.4 Mb (Figure S1D). Gene Ontology (GO) enrichment and Kyoto Encyclopedia of Genes and Genomes (KEGG) analyses showed that genes were enriched in wound healing and immune effector processes, which may relate to pigs' strong disease resistance and adaptability to harsh environments (Figure S2A, S2B). MC revealed two novel genes, *OR9G1* and *OR1A1*, which are absent from the *Sscrofa11.1* (Tables S7, S8). Given their established role in human olfaction, these genes represent promising new targets for investigating pig olfactory [10, 11]. Additionally, *UQCRFS1* is involved in the mitochondrial respiratory chain [12] and *CFAP57* is associated with multiple morphological abnormalities [13]. These pangenome genes provide insights into specific pig traits, including plateau adaptation and reproduction.

Among 23,915 gene families, 36.09% were core genes, while 32.58% were dispensable genes. Softcore families represented 30.91%, and private gene sets accounted for 0.42% (Figure 1C,D). Core genes were enriched in essential biological processes, including cellular processes, cellular development, regulation of metabolic processes, and lipid metabolic process (Figure S2C,S2D). Meanwhile, private genes showed enrichment in Estrogen signaling, Staphylococcus aureus infection, Keratinization and olfactory transduction.

### Structural variations and population genetic analysis

The Pangenie method identified 228,326 SVs (95,301 insertions (INS) and 133,025 deletions (DEL)). The average lengths are 535 and 1160 bp and the median lengths are 207 bp and 301 bp (Figure 1E). The Manta method identified 276,032 SVs (78,846 INS and 197,186 DEL). The average lengths are 206 and 1251 bp and the median lengths are 272 and 292 bp (Table S9). Unlike Manta, Pangenie identified 57,444 INS > 1 kb, demonstrating its superior detection of large INS (Figure S3A). Manta and Pangenie showed similar distributions of SVs, with most variants located in introns and intergenic regions (81.3% and 86.3%, respectively) (Figure S3B,S3C). In coding sequences, Manta identified 32,465 variants (93.2%), while Pangenie detected 5237 variants (72.1%) (Table S10). Phylogenetic analysis of Pangenie (Figure S4) revealed two major groups of pig accessions: group I (predominantly European pigs) and group II (predominantly Asian pigs); principal component analysis (PCA) and genetic structure analysis further supported this clustering (Figure 1F−I, and Figure S3D). However, the clustering results of the SVs detected by Manta are poor, highlighting the method's limitations and leading to its exclusion from further analysis (Figure S5). 1,019,330 SNPs (Figure S6) and 353,917 Indels (Figure S7) were used in population genetic analysis; the results corroborated our Pangenie findings. The SV analyses distinguished breed‐specific patterns in Asian and European pigs, highlighting their potential for identifying breed‐associated traits.

### Genome and environmental association analysis

Breeding domestic animals with climate resilience is currently an important goal for sustainable livestock production [14]. Redundancy analysis (RDA) identified 1162 SVs, 7522 SNPs, and 8126 Indels associated with 16 environmental variables (Figure S8, Table S11). SVs showed stronger environmental correlations (82.53%, exceeding 0.3) compared to SNPs (64.58%) and Indels (12.29%) (Figure 1J). UV‐B irradiance had the highest correlation with SVs (0.451), while soil silt showed the lowest correlation with Indels (0.17) (Figure 1L). We identified environment‐associated genes within 100 kb of SVs (1560), SNPs (2581), and Indels (4189). 245 shared “core adaptive genes” enriched in metabolic processes and stimulus–response regulation, suggesting their crucial role in environmental adaptation (Figure 1K, Figure S9). Mean temperature, elevation, and soil silt showed the strongest genomic associations (Figure S10A,S10B). SNPs and SVs demonstrated stronger environmental correlations compared to Indels, indicating their greater potential for studying environmental adaptation. Known high‐altitude adaptation‐related genes were identified (*KIT*, *EPAS1*, and *EGLN1*), and the newly identified gene *HAMP* (erythropoiesis and iron homeostasis) and *SYK* (fat deposition) results showed that RDA revealed significant associations between genomic variations and environmental factors. However, the numerous candidate genes identified through RDA analysis hinder the precise identification of adaptive genes.

Latent factor mixed model (LFMM) analysis identified 97 SVs, 29 SNPs, and 35 Indels (Table S12), which were associated with 116, 15, and 28 genes, respectively (Table S13). GO enrichment and PigBiobank data analyses revealed that these genes were involved in stimulus–response, cellular homeostasis, and adaptation (Figure S15). Although SVs represent less than 5% of the total variations (SNPs and Indels), SVs detected more candidate genes than SNPs and Indels. Seven genes (*ASIC2*, *ETNK2*, *MYO10*, *PTPRT*, *RETREG1*, *SOX13*, and *PLPPR1*) were detected by LFMM and RDA (SNPs, Indels, and SVs). Among these, *ASIC2* and *RETREG1* are involved in stimulus–response and stress adaptation, *PTPRT* plays a role in maintaining body size and fat deposition, and *SOX13* is crucial for early hair follicle development (Table S14). Furthermore, we identified a 73 bp INS (chromosome (chr) 9: 64,794,429 bp) significantly associated with pig adaptation to different environmental temperatures (Eco3−5) (Figure S10C, Table S12). Notably, this INS is within the intronic region of *ETNK2*, potentially regulating its expression. *ETNK2* is involved in lipid transport and metabolism, which is essential for thermoregulation (Table S14). Two genes in this candidate region, *KISS1* and *SOX13*, have been previously linked to circadian and ultradian rhythms of body temperature and hair follicle development, respectively, both crucial for temperature adaptation (Table S14). While the LFMM method currently provides limited information, it shows promising potential for accurately identifying environmentally adaptive genes (Figures S11−S14).

The combined analysis of SNPs and SVs results revealed two hotspots associated with soil silt content at 15 cm depth (Eco16) (Figure 1M, Table S15). The first hotspot (chr8: 90−92 Mb) primarily contains genes with unclear functions, which needs further investigation. The second hotspot (chr17: 38.5−41.5 Mb), identified exclusively through SV analysis, encompasses several functionally significant genes. These include *EIF6*, a metabolic regulator that drives glycolysis and fatty acid synthesis; *UQCC*, linked to height; *CEP250*, related to visual and auditory functions; and *LBP*, which influences body weight and fat storage. Additionally, *TAS2R9*, a gene for a bitter taste receptor, was identified. The genome–soil association study uncovered genes essential for pig adaptation, notably those associated with fat storage, neuromodulation, vision, hearing, and taste (Table S14).

To the best of our knowledge, such a rigorous and comprehensive analysis of pig adaptability has not been conducted to date. It may seem counterintuitive to consider pig adaptation as soil‐dependent, given that pigs use wallowing in soil primarily to regulate body temperature due to their lack of functional sweat glands [15]. However, by integrating soil data with genomic analysis, we aimed to gain novel insights into their adaptation mechanisms to high temperatures and diverse environmental conditions. Despite these findings, the absence of supporting molecular evidence underscores the need for further molecular studies to validate and expand upon these results.

### Selection signature analysis of pig body size

**FIGURE 2:**
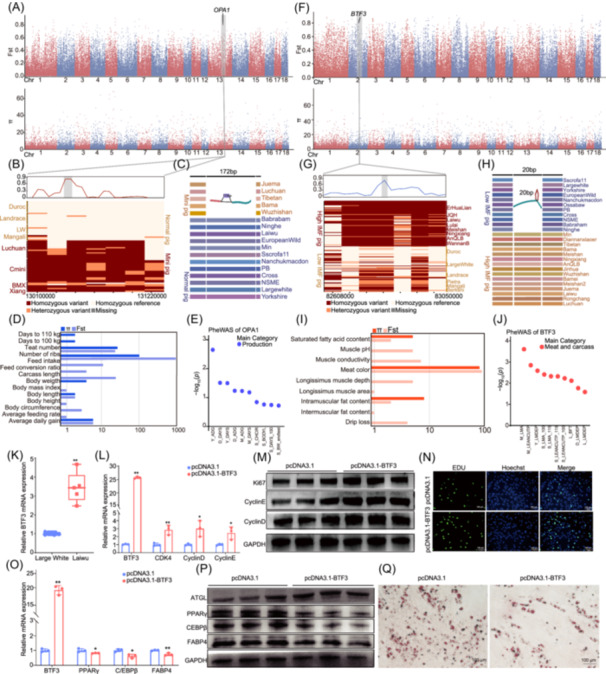
Selection signature analysis of pig body size and intramuscular fat (IMF). (A) Manhattan plots showing selected regions (Normal/Mini groups) based on the fixation index (Fst) (top) and nucleotide diversity (π) (bottom) for SVs. (B) Haplotype analysis of *OPA1*. (C) Pangenomic analysis of *OPA1*. (D) Pig quantitative trait loci (PigQTL) analysis of selected genes associated with production traits. (E) Phenome‐wide association (PheWAS) analysis of *OPA1* with the top 10 production traits. (F) Manhattan plots showing selected regions (high/low‐IMF groups) based on Fst (top) and π (bottom) for SVs. (G) Haplotype analysis of *BTF3*. (H) Pangenomic analysis of *BTF3*. (I) PigQTL analysis of selected genes associated with IMF. (J) PheWAS analysis of *BTF3* with the top 10 meat and carcass traits. (K) *BTF3* mRNA expression in the longissimus dorsi muscle of Laiwu and Large White pigs. (L) RT‐qPCR results for *BTF3*, *Ki67*, *CDK4*, and *CDK6* mRNA expression after *BTF3* overexpression. (M) Western blot analysis showing the protein expression of *Ki67*, *CyclinD*, and *CyclinE* after *BTF3* overexpression. (N) EdU staining to detect the proliferation of primary pig IMF cells after BTF3 transfection. Cells in the S phase were stained with EdU (green), while nuclei were stained with Hoechst (blue) and counted using ImageJ. (O) RT‐qPCR analysis showed the mRNA expression levels of *BTF3*, *PPAR*γ, C/*EBP*β, and *FABP4* after *BTF3* overexpression. (P) Western blot analysis detected the protein expression of differentiation‐related genes following *BTF3* overexpression. (Q) Oil Red O staining was performed to detect lipid accumulation following *BTF3* overexpression, indicating the formation of lipid droplets. The above results were representative of means ± SD of three independent experiments. Student's *t*‐test was used to determine significance *p < 0.05; **p < 0.01.

Pigs are valuable resources due to their efficient meat production, enabled by their size and their potential medical applications. We analyzed 87 pig accessions, categorizing them into Normal and Mini groups (Table S16). Fixation index (Fst) analysis of SVs revealed average and maximum values of 0.13 and 0.90, respectively, with the top 1% threshold at 0.56 (Figure S16A, Table S17). Within the highest selective region (chr5: 85.6−85.65 Mb), we identified *TRNAD‐GUC* and *MIR135‐2*, suggesting potential roles of transfer‐RNA and micro‐RNA in skeletal development. *CDKAL1*, which showed the second highest Fst value (0.89), has previously been linked to body mass regulation. Other key genes identified included *PTPRT* (associated with feed intake and size), *PLPPR1* (involved in phospholipid metabolism), and *NFIA* (implicated in glucose homeostasis and skeletal development). Nucleotide diversity (π) analysis showed lower diversity in the Normal group (9.3 × 10^−6^) compared to the Mini group (9.6 × 10^−6^) (Figure S16B). Interestingly, genes like *APLP2* and *PTPRT* were detected by both methods (Table S18). Pig quantitative trait loci (PigQTL) (Figure 2D), GO enrichment, and PigBiobank data revealed that these genes were associated with growth and developmental processes (Figure S16C,S16D and Figure S17). We identified 36 colocalized selective regions, GO enrichment indicating these gene associations with ossification and bone trabecula formation (Table S19). Notably, *OPA1* showed differences between Normal and Mini pigs (Figure 2A−C). PigBiobank analyses further validated *OPA1* associations with body weight and feed intake (Figure 2E and Figure S16E).

### Selection signature and function analysis of IMF

156 pig samples were classified into high‐ and low‐IMF groups according to the public resources (Table S16). Analysis of high‐ and low‐IMF groups revealed significant genetic differentiation (average Fst = 0.16, max = 0.92) (Figure S18A). Among the genes with high Fst values, *KIT*, traditionally linked to coat color, was also associated with fresh meat color and overall meat quality. Additional significant genes included *WWOX* (linked to lipid metabolism), *SND1* (involved in cholesterol regulation), and *ROCK1* (influencing myogenesis and meat quality) (Table S14). π was notably higher in the high‐IMF group (π = 9.6 × 10⁻⁶) than in the low‐IMF group (π = 7.9 × 10⁻⁶) (Figure S18B). We identified seven colocated selective regions through Fst and π analyses, primarily associated with lipid transport and localization (Table S19). Further pangenome and haplotype analyses of *WWOX* and *SND1* (Figure S18C–D,S18D) highlighted that these genes may influence IMF deposition (Figure S19C,S19D). GO enrichment, PigBiobank, and PigQTL results showed that these genes were associated with meat quality traits (Figure 2I, Figure S19A,S19B, and S20). Expression analysis of 12 selected genes showed significant differential expression between high‐ and low‐IMF samples (*p* < 0.05) (Figure S21A), supporting their potential regulatory roles in IMF development.

*BTF3*, identified in Fst analysis (0.81) (Figure 2F), showed a distinct haplotype between Asian and European pigs (Figure 2G), with a 414 bp variation upstream of its CDS, suggesting that a 20 bp deletion event might influence IMF differences between Asian and European pigs (Figure 2H). The PigBiobank database demonstrated that *BTF3* is associated with meat quality traits across diverse pig populations, indicating its potential as a key regulator of IMF deposition (Figure 2J, Figure S19E). *BTF3* showed significantly higher expression in high‐IMF pigs compared to low‐IMF pigs (*p* < 0.05) (Figure 2K). To investigate *BTF3*'s role in intramuscular adipocyte proliferation and differentiation, we generated overexpression (pcDNA3.1‐*BTF3*) and inhibition (si‐*BTF3*) plasmids.

*BTF3* overexpression significantly increased the expression of proliferation marker genes (*p* < 0.05) (Figure 2L,M and Figure S21B), whereas its inhibition led to significant decreases (*p* < 0.01) (Figure S21D,S21E), indicating that *BTF3* promotes adipocyte proliferation. CCK‐8 assays confirmed that *BTF3* overexpression enhances adipocyte proliferation, with the opposite effect observed upon *BTF3* inhibition (Figure S21C,S21F). Additionally, EdU incorporation assays demonstrated that *BTF3* overexpression promotes adipocyte proliferation, while its interfered inhibits adipocyte proliferation (Figure 2N, Figure S21G,S21H). In contrast, analysis of adipogenic differentiation markers showed that *BTF3* overexpression significantly reduced their expression (*p* < 0.05) (Figure 2O,P), while inhibition increased it (*p* < 0.05) (Figure S21I,S21G, and S21K). Oil Red O staining further validated that *BTF3* overexpression inhibits lipid droplet formation, while its suppression promotes lipid accumulation (Figure 2Q, Figure S21L). These findings suggest that *BTF3* enhances cell proliferation while inhibiting differentiation in pig intramuscular adipocytes, underscoring its pivotal role in IMF deposition. While we identified *BTF3*'s association with IMF, its regulatory mechanism remains to be elucidated. We propose that *BTF3* plays dual roles in adipogenesis: promoting early adipose tissue proliferation while preventing excessive fat accumulation through negative feedback when overexpressed. This balance suggests that *BTF3* maintains adipogenic potential while preserving cellular pluripotency, though its precise regulatory mechanisms warrant further investigation.

While pangenome analysis highlights SVs' importance in identifying candidate genes [16], our pipeline has limitations in analyzing complex SVs [17], particularly in regions with segmental duplications, tandem repeats, and copy number variations [18]. Although effective for SV genotyping within the constructed graph, its accuracy lags behind SNP and Indel detection. High‐quality genome assemblies currently offer better complex SV detection, but emerging unified pangenome tools and reference‐grade assemblies should address these limitations. In conclusion, our study demonstrates the prevalence of SVs in pig genomes and their utility in population genetics, and trait association studies, providing a valuable resource for genetic improvement and biological discovery.

## METHODS

Detailed procedures for biological sample collection, sequencing protocol, data processing techniques for sequencing data, and bioinformatic and statistical analysis approaches are available in the Supplementary Information.

## AUTHOR CONTRIBUTIONS

**Dong Li**: Investigation; formal analysis; writing—review and editing. **Yulong Wang**: Writing—review and editing; investigation; formal analysis. **Tiantian Yuan**: Writing—review and editing; visualization; investigation. **Minghao Cao**: Writing—review and editing; investigation; formal analysis. **Yulin He**: Validation. **Lin Zhang**: Visualization; investigation. **Xiang Li**: Investigation; formal analysis. **Yifan Jiang**: Investigation. **Ke Li**: Visualization; investigation. **Jingchun Sun**: Investigation. **Guangquan Lv**: Formal analysis. **Guosheng Su**: Project administration. **Qishan Wang**: Project administration. **Yuchun Pan**: Project administration. **Xinjian Li**: Project administration. **Yu Jiang**: Project administration. **Gongshe Yang**: Project administration. **Martien A M Groenen**: Project administration. **Martijn F. L. Derks**: Writing—review and editing; supervision; conceptualization. **Rongrong Ding**: Methodology; conceptualization; project administration; and supervision. **Xiangdong Ding**: Supervision; project administration; conceptualization; resources. **Taiyong Yu**: Project administration; funding acquisition; conceptualization; supervision; resources.

## CONFLICT OF INTEREST STATEMENT

The authors declare no conflicts of interest.

## Supporting information


**Figure S1.** Pig genome assembly and pangenome analyses.
**Figure S2.** Gene enrichment analysis of pig pangenome genes.
**Figure S3.** Results of structural variation (SV) analysis.
**Figure S4.** Analysis of the population structure of Pangenie's SVs.
**Figure S5.** Analysis of the population structure of Manta's SVs.
**Figure S6.** Analysis of the population structure of single‐nucleotide polymorphisms (SNPs).
**Figure S7.** Analysis of the population structure of insertions/deletions (Indels).
**Figure S8.** Correlation analysis of 16 climate variables with “|r^2^| < 0.7”.
**Figure S9.** Gene Ontology (GO) enrichment of “core adaptive genes”.
**Figure S10.** Pig genome–environment association analyses.
**Figure S11.** Latent factor mixed model (LFMM) analysis of Eco1‐Eco4.
**Figure S12.** LFMM analysis of Eco5‐Eco8.
**Figure S13.** LFMM analysis of Eco9‐Eco12.
**Figure S14.** LFMM analysis of Eco13‐Eco16.
**Figure S15.** Enrichment analyses of adaptive genes.
**Figure S16.** Selection signature analysis of pig body size.
**Figure S17.** Phenome‐wide association (PheWAS) analysis of genes enriched in growth‐related pathways with production traits.
**Figure S18.** Selection signature analysis of intramuscular fat (IMF).
**Figure S19.** Analysis of candidate genes associated with IMF.
**Figure S20.** PheWAS analysis of genes enriched in lipid metabolic process based on meat and carcass traits.
**Figure S21.** Functional analysis of IMF‐associated candidate genes.

**Table S1.** Summary statistics of the pig genome assemblies in this study.
**Table S2.** Summary statistics of repeat analyses for 27 genomes.
**Table S3.** Summary statistics of gene annotation and BUSCO evaluation annotation (vertebrata_odb10) of 26 pig genomes and the reference genome.
**Table S4.** Summary statistics of non‐coding RNAs in genomes.
**Table S5.** Profiles of the pig pangenomes constructed from autosomal sequences of 27 assemblies.
**Table S6.** Analysis of different methods of pig pangenome sequence.
**Table S7.** Summary statistics of annotation analysis of different methods of pig pangenome sequence.
**Table S8.** Analysis of different methods of pig pangenome sequence for genes.
**Table S9.** Summary statistics of different methods of structural variations (SVs).
**Table S10.** Summary statistics of Ensemble Variant Effect Predictor result of SVs.
**Table S11.** Environmental variables used in this study and redundancy analysis (RDA) results for different variations with different variables.
**Table S12.** Summary of latent factor mixed model (LFMM) significant sites with gene information after q‐value correction at 1%.
**Table S13.** Summary of candidate genes of LFMM analysis.
**Table S14.** Literature references for the genes mentioned in this paper.
**Table S15.** Summary of candidate genes of single‐nucleotide polymorphisms (SNPs) and SVs for Eco16 of LFMM analysis in two hotspots.
**Table S16.** Individual group information for selection signature analysis.
**Table S17.** Genes located in the top 1% high fixation index (Fst) value regions of pig body size and intramuscular fat (IMF).
**Table S18.** Genes located in the top 1% high nucleotide diversity (π) value regions of pig body size and IMF.
**Table S19.** Candidate functional categories of the genes located in top 1% high Fst and π value regions of pig body size and IMF.
**Table S20.** Summary of sequencing data used for Bamei (BM), Hanjiang Black (HJB), Juema (JM), and Large White (LW) genome de novo assembly.
**Table S21.** PacBio Iso‐Seq and RNA transcripts used for Bamei and Juema genome annotation.
**Table S22.** NCBI proteins used in BRAKER2.
**Table S23.** Geographical origin and sequencing data statistics of 603 pigs used in the study.
**Table S24.** Environmental variable selection from different databases.
**Table S25.** Pig breed phenotypes for Worldclim, Soil, and latitude.
**Table S26.** Pig breed phenotypes.
**Table S27.** Primers used for quantitative PCR.

## Data Availability

The PacBio HiFi reads, Hi‐C reads, PacBio Iso‐Seq reads, and Illumina paired‐end reads sequencing data have been deposited in NCBI under BioProject PRJNA858995, PRJNA980289, and PRJNA975220. The raw sequencing data generated for the 10 genomes have been submitted to the GSA under accession number PRJCA005901. The assembled Bamei, Juema, Hanjiang Black, and Large White pig genomes can be obtained from the GenBank under the numbers GCA_030704935.1, GCA_040869115.1, GCA_044906185.1, and GCA_044906105.1. The data and scripts used are saved in GitHub https://github.com/ld9866/Pig_pangenome_iMeta. The pangenome, annotation, and SV‐Panel in this study are freely available at http://animal.omics.pro/code/index.php/panPig. Supplementary materials (figures, tables, graphical abstracts, slides, videos, Chinese translated versions, and update materials) can be found in the online DOI or iMeta Science http://www.imeta.science/.
