# Supplementary material for: Pangenome and genome variation analyses of pigs unveil genomic facets for their adaptation and agronomic characteristics
Source: Imeta. 2024 Dec 26;3(6):e257. doi: 10.1002/imt2.257 (PMC11683468; doi:10.1002/imt2.257)

Supporting Information

Pangenome and genome variations analysis of pigs unveil genomic facets for its adaptation and agronomic characteristics.

**Running title**: Genomic insights into pig adaptation and agronomic traits

Dong Li^1 #^, Yulong Wang^1 #^, Tiantian Yuan^1 #^, Minghao Cao^1 #^, Yulin He^1^, Lin Zhang^1^, Xiang Li^1^, Yifan Jiang^2^, Ke Li^3^, Jingchun Sun^4^, Guangquan Lv^1^, Guosheng Su^5^, Qishan Wang^6^, Yuchun Pan^6^, Xinjian Li^7^, Yu Jiang^1^, Gongshe Yang^1^, Martien A.M. Groenen^8^, Martijn F. L. Derks^8^ ^*^, Rongrong Ding^1 *^, Xiangdong Ding^2 *^, Taiyong Yu^1 *^

^1^ Key Laboratory of Animal Genetics, Breeding and Reproduction of Shaanxi Province, Laboratory of Animal Fat Deposition & Muscle Development, College of Animal Science and Technology, Northwest A&F University, Yangling, Shaanxi 712100, China.

^2^ State Key Laboratory of Animal Biotech Breeding National Engineering Laboratory for Animal Breeding, College of Animal Science and Technology, China Agricultural University, Beijing 100193, China

^3^ Key Laboratory of Vertebrate Evolution and Human Origins, Chinese Academy of Sciences, Beijing 100044, China

^4^ Institute of Subtropical Agriculture, Chinese Academy of Sciences, Changsha, Hunan 410125, China

^5^ Centre for Quantitative Genetics and Genomics, Aarhus University, Aarhus, DK-8830, Denmark

^6^ Department of Animal Science, College of Animal Science, Zhejiang University, Hangzhou 310058, China

^7^ Sanya Institute, Hainan Academy of Agricultural Science, Sanya, Hainan 572000, China

^8^ Animal Breeding and Genomics, Wageningen University and Research, Wageningen, 6708PB, The Netherlands

^#^These authors contributed equally: Dong Li, Yulong Wang, Tiantian Yuan, Minghao Cao

^*^Correspondence: [yutaiyong310@nwsuaf.edu.cn](mailto:yutaiyong310@nwsuaf.edu.cn) (Taiyong Yu); [xding@cau.edu.cn](mailto:xding@cau.edu.cn) (Xiangdong Ding); [dingrongrong92@nwafu.edu.cn](mailto:dingrongrong92@nwafu.edu.cn) (Rongrong Ding); martijn.derks@wur.nl (Martijn F. L. Derks)

**Supplementary materials and methods**

## Sample collection

High-quality genome assemblies and representative individuals are fundamental for studying genomic variation and constructing comprehensive pangenomes. Our selection of breeds addresses several key gaps in existing pig genomic resources. The previously available Bamei (BM) pig genome was limited to scaffold-level assembly (contig N50 = 70.9 kb, GenBank: GCA_001700235.1), lacking chromosome-level resolution. Despite its global commercial significance, the Large White (LW) breed lacked a high-quality reference genome, with existing assemblies restricted to scaffold level and contig N50 values below 10Mb (GenBank: GCA_029784025.1, GCA_029890215.1, GCA_029890225.1, GCA_001700135.1). Importantly, Hanjiang Black (HJB) and Juema (JM) pigs, two significant indigenous breeds, had no previously reported genome assemblies. Our strategic selection of BM, HJB, and JM pigs significantly enhances the pangenome's representation of Northwest China's indigenous genetic diversity. The high-quality Large White pig genome also contributes valuable genomic information for commercial breed research.

Blood samples were collected from the Bamei Pig Breeding Farm in Qinghai, Province, the Hanjiang Black Pig Breeding Farm in Shaanxi Province, and the Juema Pig Breeding Farm in Gansu Province, China. The Large White pig sample was collected from the Shunxin Pig Breeding Farm in Shaanxi Province, China.

## Pig sequencing and genome assembly

Genomic DNA was extracted from the blood of each accession. For circular consensus sequencing (CCS), SMRTbell libraries (15 kb) were constructed according to the protocol released from PacBio and sequenced on the PacBio Sequel II platform to generate HiFi reads. The High-throughput Chromosome Conformation Capture (Hi-C) libraries were controlled for quality and sequenced on an Illumina NovaSeq 6000 platform.

A total of 4 pig accessions, BM, JM, HJB, and LW were sequenced in our study **(Table S20)**. Primary assemblies were generated from Hifiasm (v: 0.15.5-r350) with the default parameters [1]. For the BM and JM genomes, the Hi-C data were used to obtain a chromosome-level assembly, the quality control processes were processed with fastp (v: 0.20) [2] and the chromosome-length genome was produced using the Juicer (v: 1.6) and 3D-DNA (v: 201008) (--merge 0) with the default parameters to generate a Hi-C contact matrix at a fine resolution using default parameter (a detailed description of the pipelines: http://aidenlab.org/assembly/manual_180322.pdf). The results were polished using the Juicebox Assembly Tools (v: 1.11.8) for visualization of Hi-C maps and genome assembly correction [3-5]. The HJB, LW, and 10 genomes from our previous study [6] were anchored and oriented to chromosomes using the reference-guided software RagTag (v: 2.1) with default parameter [7] using *Sscrofa*11.1 as reference, and the genome quality was evaluated using the benchmarking universal single-copy orthologs (BUSCO) (v: 5.0.2) (vertebrata_odb10s) [8].

## Genome annotation

For the pig genome annotation, the RepeatModeler (v: 2.0.2) and RepeatMasker (v: 4.1.2-p1) were used to screen the repetitive sequences [9; 10].

The BRAKER1 (v: 2.1.6) [11] generated a gene prediction set supported by both RNA-seq and homologous protein evidence for BM and JM. Illumina RNA-Seq reads were aligned to masked assemblies using STAR (v: 2.7.3a55) [12] **(Table S21)** and were used as transcript evidence in the BRAKER1 pipeline. We used OrthoDB (Vertebrata) and 9 species annotations **(Table S22)** from NCBI for the protein homology prediction through the BRAKER2 (v: 2.1.6) pipeline for genomes [13]. PacBio Isoseq reads were analyzed following the IsoSeq3 pipeline (github.com/PacificBiosciences/IsoSeq), and the scripts from Cupcake (v: 29.0) and GeneMarkS-T (v: 1.14) were used to predict protein-coding regions in the transcripts. Finally, the long-read version of TSEBRA (v: 1.0.3) combined the three gene sets using all extrinsic evidence to generate gene predictions [14]. The transanno (v: 0.4.3) was used to generate gene sets for the genome assemblies (github.com/informationsea/transanno). Briefly, minimap2 (v: 2.26) [15] was used to create a chain file for query_fasta and pig reference (*Sscrofa*11.1) and transfer the GFF3 (Ensembl Release 110) to genomes. Finally, the modules agat_sp_ensembl_output_style.pl and agat_sp_merge_annotations.pl from AGAT (v: 1.2.0) (github.com/NBISweden/AGAT) were used to combine the gene evidence to the final annotation, and the final genome annotation was assessed by BUSCO (v: 5.0.2) (vertebrata_odb10s) [8].

Non-coding RNA genes (ncRNA) were annotated using Rnammer (v: 1.2) [16], tRNAScan-SE (v: 2.0.9) [17] and Infernal (v: 1.1.4) [18] based on the Rfam database (release 14.7) [19].

Functional annotation was achieved by comparing predicted proteins against public databases with an E-value threshold of 1e-5, including eggNOG-mapper (v: 2.1) [20], NCBI non-redundant protein sequences database (Nr) (ftp.ncbi.nlm.nih.gov/blast/db/FASTA/), SwissProt (http://www.gpmaw.com/html/swiss-prot.html) [21], and TrEMBL (http://www.uniprot.org) using Diamond BLASTP (v: 2.0.11) [22]. The final functional annotation results retained only high-quality protein-coding genes, defined by an identity threshold greater than 85% and a bitscore exceeding 200.

## The graph pangenome constructions

A total of 27 pig accessions were used in our pangenome construction **(Table S1)** and only the autosomal sequences were retained for subsequent analysis. Minigraph-Cactus (MC) could build graphs containing all forms of genetic variation while allowing the use of current mapping and genotyping tools which have shown good performance in humans [23; 24], plants [25], and animals [26]. We used MC (v: 2.7) [27] to construct the pig graph pangenome, and the additional sequence analysis followed Edward’s study [26]. The vg (v: 1.5.3) [28] was used to extract the non-reference sequence from MC. In brief, the longest allele sequence of each variation site was extracted and stored in the FASTA format. The genetic distance among these assemblies was estimated using Mash (v: 2.3) [29]. We downloaded the pig pangenome sequences from Tian [30], Li [31], and Du [32] studies. The RepeatMasker (v: 4.1.2-p1) and RepeatModeler (v: 2.0.2) [9; 10] were used to study the repetitive sequences and we used the BlastX (v: 2.0.11) to compare with the NCBI non-redundant protein sequences database (Nr)(ftp.ncbi.nlm.nih.gov/blast/db/FASTA/) (identify 80%; similarity 80%).

## The graph pangenome analysis

The core and dispensable gene sets were defined based on gene family clustering using OrthoFinder2 (v: 2.5.4) [33]. We classified the gene sets into four categories: core gene family, softcore gene family, dispensable gene family, and private gene family. Gene families that were shared among all accessions were defined as core gene families, those that were absent in one or two accessions were defined as softcore gene families, those that were absent in more than two accessions were defined as dispensable gene families, and those that were present in only one accession were defined as private gene families. For gene function annotation, Gene Ontology (GO) and Kyoto Encyclopedia of Genes and Genomes (KEGG) analyses were performed using g: Profiler (v: 2023_update) [34].

## Read processing

For the next-generation sequencing (NGS), the adapters and low-quality reads were filtered away using the fastp (v:0.20) [2] with default parameters and then mapped to the Duroc pig reference genome (*Sscrofa*11.1) using NVIDIA Parabricks (4.0.1-1) (germline caller, HaplotypeCaller) [35]. The GVCF files were merged for joint variant calling with GLnexus (v: 1.4.1) [36]. Manta (v: 1.6) [37] was used to detect structural variation (SVs) according to the manual with default parameters. The short reads from 598 pig accessions **(Table S23)** were mapped to the MC pig-pangenome following the Pangenie (v: 3.0.1) pipeline [38]. To comprehensively investigate SVs within and among 81 pig populations, we employed the Pangenie and Manta pipeline to genotype 598 short-read samples, focusing on insertions (INS) and deletions (DEL) ranging from 50 bp to 100 kb for subsequent population genetic analyses.

## Population structure and phylogenetic analyses

To evaluate the impact of our SV sets on population genetic analyses, we compared them with single-nucleotide polymorphisms (SNPs) and small insertions / deletions (Indels) data across 598 pig accessions and 5 outgroup samples collected from diverse geographic regions. These samples were sequenced to an average depth of 14.9X, with a 99.3% mapping ratio to the reference genome (**Table S23**). After quality control (genotyping rate > 0.9, minor allele frequency > 0.01), 115,043 SVs genotyped by Pangenie were used for analysis. Using relaxed quality control parameters (genotyping rate > 0.9, minor allele frequency > 0.01), the Manta method identified 50,502 SVs.

We used PLINK (v: 1.9) to calculate the average shared allele distance matrix between individuals (--distance-matrix) [39]. The Principal component analysis (PCA) was performed using PLINK (module: PCA), and the Neighbor-Joining (NJ) tree was constructed using MEGA-X (v: 10.0.5) [40] and visualized with iTOL (v: 6.5.8) [41]. The population genetic structure was examined using the Admixture (v: 1.23) [42] with K values (the putative number of populations) from 2 to 10. LDblockshow (v: 1.4) [43] was used to visualize linkage disequilibrium and haplotype block regions. Ensemble Variant Effect Predictor (VEPtools v: 111) [44] was used to predict the effects of the SVs identified by the Manta and Pangenie method.

## Environmental Variable Selection and Genome Environment Association Analyses

For each population, we recorded a single central coordinate of the village or city. The climatic data were characterized using the 19 Bioclim variables at 30 arcsec resolution from the WorldClim v2 database [45]. The levels of UV-B irradiance were extracted from the glUV dataset with a spatial resolution of 15 arc-minutes [46]. The physical and chemical properties of the soils were obtained from SoilGrids250m [47] and the elevation data were acquired from Amazon Web Services Terrain Tiles web services (arn:aws:s3:::elevation-tiles-prod), a total of 61 environmental variables were obtained from these databases **(Table S24)**.

To address the issue of multicollinearity, which can lead to less accurate predictions [48], we calculated correlation coefficients among the 61 environmental variables using the R package "psych" (v: 2.2.5) [49]. Since redundancy analysis (RDA) is a regression-based method, and so can be subject to problems when using highly correlated predictors. Following Dormann's recommendation [48], we removed highly correlated predictors by eliminating variables with |r| > 0.7. This process resulted in the retention of 16 factors for subsequent genome-environment association (GEA) analysis (**Table S25**). After filtering location data and performing quality control, we retained 65,536 SVs, 1,564,095 SNPs, and 1,428,904 Indels from 552 samples.

The genome-environment association (GEA) analysis was conducted using two complementary methods: RDA and latent factor mixed models (LFMM) [50]. RDA is a two-step analysis in which genetic and environmental data are analyzed using multivariate linear regression, producing a matrix of fitted values. Then PCA of the fitted values is used to produce canonical axes, which are linear combinations of the predictors. RDA was performed using the RDA function in the vegan package (v: 2.6-4) [51]. Following Forester's approach [52], significant environment-associated variants were identified as those with loadings in the distribution tails, using a standard deviation cutoff of 3 astandard deviation (two-tailed *p*-value = 0.0027) (more detailed methods are available at: https://popgen.nescent.org/2018-03-27_RDA_GEA.html). LFMM analysis was conducted according to Nocchi's pipeline [53] using the LEA package (v:3.0) [54]. The candidate adaptive variations were selected based on an FDR < 1% according to the recommended parameters (github.com/GabrieleNocchi/betula_platyphylla_local_adaptation). For both methods, genes within a 100 kb window upstream and downstream of significant loci were designated as environment-associated genes.

## Phenotypic characterization of pig populations and selection signature analysis

The database of phenotypic traits of European and Asian local pig populations was used to determine global breed differences in phenotype. In brief, we collected the body size and IMF of each population according to Animal Genetic Resources in China: Pigs [55], European local pig breeds-diversity and performance [56], and related publications **(Table S26)**.

The fixation index (Fst) and Nucleotide diversity (π) were calculated by vcftools (v: 0.1.16) with sliding windows of 50 kb with a step size of 25 kb. The top 1% regions were assigned to candidate selective regions, and genes in these regions were considered as candidate genes, and the Phenotype-associated loci derived from the pigQTLdb (April 25th, 2023) [57] were partitioned into quantitative trait loci (QTL) regions. Functional predictions of candidate genes were conducted using the PigBiobank [58] database.

## Real-time quantitative PCR (RT-qPCR)

Laiwu Black pig, a Chinese indigenous breed primarily distributed in Shandong Province, is renowned for its excellent meat quality and high intramuscular fat content. Studies have shown that its IMF content ranges between 10%−13%, making it the highest among known Chinese indigenous pig breeds [59]. Large White typically has IMF content ranging from 1% to 3%. Total RNA was extracted from the Large White and Laiwu Black longissimus dorsi (100 kg), primary adipocyte cells using TRIzol reagent (Takara Bio, Otsu, Japan) according to the manufacturer's instructions. The RNA was then reverse-transcribed into cDNA using the PrimeScript RT Reagent Kit (Takara Bio, Otsu, Japan). RT-qPCR was performed using the SYBR Premix Ex Taq Kit (Vazyme Biotech, Nanjing, China). The primers used for quantitative PCR are listed in **Table S27**.

## Pig Primary Adipocyte Culture

Pig primary adipocytes were isolated from the longissimus dorsi tissue at the back of 3-day piglets. Primary adipocyte cells were isolated and cultured as described previously [60]. Briefly, longissimus dorsi muscle was minced and digested into 1 mm³ pieces and digested with 0.2% type 1 collagenase (270 U/mg; Gibco, Carlsbad, CA, USA) in a water bath shaker at 37°C for 2 hours. The digestion was stopped by adding fetal bovine serum (Gibco, Grand Island, NY, USA). The samples were filtered through 70-mesh and 200-mesh sieves, and the filtrate was centrifuged at 1,500 rpm for 10 minutes. After the supernatant was discarded**, the cells were** washed with serum-free DMEM/F12 medium (DMEM/F12; Hyclone, Logan, UT, USA) and cultured in growth medium containing DMEM/F12 supplemented with 10% fetal bovine serum on collagen-coated plates at 37°C in 5% CO₂. Non-attached cells were discarded after 1.5 hours**, and** the culture medium was changed every 2 days thereafter. Upon reaching 100% confluence, the cells were maintained in growth medium for two days and then cultured for 2 days in DMEM/F12 supplemented with 1 μM dexamethasone (DEX, Solarbio, Beijing, China), 0.5 mM 3-isobutyl-1-methyl xanthine (IBMX, Sigma, St. Louis, MO, USA), and 10 μg/mL insulin (Sigma, St. Louis, MO, USA). Cell differentiation was then maintained in DMEM/F12 medium supplemented with 10 μg/mL insulin for 4 days.

## Overexpression vector and small interfering RNA transfection

The BTF3-coding sequence (CDS) expression plasmid was generated by cloning the full-length open reading frame of the *BTF3* gene (NM_001244998.1) into the pcDNA3.1 mammalian expression vector (Tsingke Biotechnology, Beijing, China). The siRNA and negative control (NC) were also obtained from Tsingke Biotechnology (Beijing, China). For proliferation studies, pig primary adipocytes were transfected with ~4 μg plasmid using 4 μL Lipofectamine 2000 (Invitrogen, USA) and 50 nM siRNA or NC per well in a 6-well plate. Cells were harvested 24 h post-transfection. For differentiation studies, transfection was performed at 80% confluence. When cells reached confluency, the growth medium was replaced with differentiation medium, and cells were harvested after 6 days of differentiation.

## Western blotting

Proteins were extracted from cells using radioimmunoprecipitation assay (RIPA) buffer with 1% (v/v) reverse transcription kits (Cwbio, China). The total protein sample was separated in the SDS-polyacrylamide gel. Then, it was transferred into a PVDF membrane (Millipore, Bedford, MA, USA). Next, the membrane was blocked in 5% defatted milk for 2 h. The primary antibody 4 was incubated overnight. The antibodies used included Ki67 (1:1000; Abcam; Britain), CyclinD (1:1000; ProteinTech; China), CyclinE (1:1000; ProteinTech; China), ATGL(1:1000; Abcam; Britain), PPARγ (1:1000; ProteinTech; China), C/EBPβ (1:1000; Abcam; Britain), FABP4 (1:1000; ProteinTech; China), and GAPDH (1:2000; ProteinTech; China). After incubation, the membrane was washed three times with TBST solution, and secondary antibodies (Goat Anti-Mouse IgG, Boster, BA1038; Goat Anti-Rabbit IgG, Boster, BA1039) were added. Finally, the western blots were exposed to the Bio-Rad imaging system. All protein levels were normalized to that of the glyceraldehyde-3-phosphate dehydrogenase (GAPDH), and densitometric quantification of the western blotting bands was performed using ImageJ (v:1.53) software.

## Ethynyl-20-Deoxyuridine (EdU) Assay

Pig primary adipocytes were seeded into 48-well cell culture plates, and transfections were carried out once the cell density reached 30%−40%. After 24 hours of transfection, the cells were processed following the instructions of the Cell-LightTM EdU Apollo567 In Vitro Kit (RiboBio, Guangzhou, China). Subsequently, the cells were captured under a fluorescence microscope.

## CCK-8 Assay and Oil-red O staining

Pig primary adipocytes were plated in 96-well cell culture plates. Transfection was conducted when the cell density reached 30%−40%. After 24 hours, 10 μL of Cell-Counting Kit-8 (CCK-8) reagents (Solarbio, Beijing, China) were added to the cells for a 2-hour incubation period. Subsequently, the absorbance of the cells at 450nm was measured using an enzyme-labeled instrument, and the data were subjected to statistical analysis. After 8 days of differentiation in pig primary adipocytes, respectively, differentiated adipocytes were washed three times with PBS and fixed for 30 minutes in 4% polyformaldehyde. Cells were then stained with an Oil-red O working solution for 1 hour according to the manufacturer's instructions. Then cells were washed four times with PBS and photographed under a microscope.

## Statistical analyses

The differences between groups were analyzed using unpaired or paired Student's t-test, and the data are presented as mean ± standard deviation (SD). Statistical significance was set at * *p* < 0.05 and ** *p* < 0.01. For multiple comparisons, statistical significance was determined using post-hoc analysis with Bonferroni correction. Adjusted *p*-values were considered significant at * *p* < 0.05 and ** *p* < 0.01.

**References**

1. Cheng, Haoyu, Gregory T Concepcion, Xiaowen Feng, Haowen Zhang, Heng Li. 2021. “Haplotype-resolved de novo assembly using phased assembly graphs with hifiasm.” *Nature Methods* 18: 170-175. https://doi.org/10.1038/s41592-020-01056-5

2. Chen, Shifu, Yanqing Zhou, Yaru Chen, Jia Gu. 2018. “fastp: an ultra-fast all-in-one FASTQ preprocessor.” *Bioinformatics* 34: i884-i890. https://doi.org/10.1093/bioinformatics/bty560

3. Durand, Neva C, Muhammad S Shamim, Ido Machol, Suhas SP Rao, Miriam H Huntley, Eric S Lander, Erez Lieberman Aiden. 2016. “Juicer provides a one-click system for analyzing loop-resolution Hi-C experiments.” *Cell Systems* 3: 95-98. https://doi.org/10.1016/j.cels.2016.07.002

4. Durand, Neva C, James T Robinson, Muhammad S Shamim, Ido Machol, Jill P Mesirov, Eric S Lander, Erez Lieberman Aiden. 2016. “Juicebox provides a visualization system for Hi-C contact maps with unlimited zoom.” *Cell Systems* 3: 99-101. https://doi.org/10.1016/j.cels.2015.07.012

5. Dudchenko, Olga, Sanjit S Batra, Arina D Omer, Sarah K Nyquist, Marie Hoeger, Neva C Durand, Muhammad S Shamim, Ido Machol, Eric S Lander, Aviva Presser Aiden. 2017. “De novo assembly of the Aedes aegypti genome using Hi-C yields chromosome-length scaffolds.” *Science* 356: 92-95. https://doi.org/10.1126/science.aal3327

6. Jiang, Yi-Fan, Sheng Wang, Chong-Long Wang, Ru-Hai Xu, Wen-Wen Wang, Yao Jiang, Ming-Shan Wang, Li Jiang, Li-He Dai, Jie-Ru Wang. 2023. “Pangenome obtained by long-read sequencing of 11 genomes reveal hidden functional structural variants in pigs.” *iScience* 26: https://doi.org/10.1016/j.isci.2023.106119

7. Alonge, Michael, Sebastian Soyk, Srividya Ramakrishnan, Xingang Wang, Sara Goodwin, Fritz J Sedlazeck, Zachary B Lippman, Michael C Schatz. 2019. “RaGOO: fast and accurate reference-guided scaffolding of draft genomes.” *Genome Biology* 20: 1-17. https://doi.org/10.1186/s13059-019-1829-6

8. Manni, Mosè, Matthew R Berkeley, Mathieu Seppey, Felipe A Simão, Evgeny M Zdobnov. 2021. “BUSCO update: novel and streamlined workflows along with broader and deeper phylogenetic coverage for scoring of eukaryotic, prokaryotic, and viral genomes.” *Molecular Biology and Evolution* 38: 4647-4654. https://doi.org/10.1093/molbev/msab199

9. Chen, Nansheng. 2004. “Using Repeat Masker to identify repetitive elements in genomic sequences.” *Current Protocols in Bioinformatics* 5: 4.10. 11-14.10. 14. https://doi.org/10.1002/0471250953.bi0410s05

10. Flynn, Jullien M, Robert Hubley, Clément Goubert, Jeb Rosen, Andrew G Clark, Cédric Feschotte, Arian F Smit. 2020. “RepeatModeler2 for automated genomic discovery of transposable element families.” *Proceedings of the National Academy of Sciences* 117: 9451-9457. https://doi.org/10.1073/pnas.1921046117

11. Hoff, Katharina J, Simone Lange, Alexandre Lomsadze, Mark Borodovsky, Mario Stanke. 2016. “BRAKER1: unsupervised RNA-Seq-based genome annotation with GeneMark-ET and AUGUSTUS.” *Bioinformatics* 32: 767-769. https://doi.org/10.1093/bioinformatics/btv661

12. Dobin, Alexander, Carrie A Davis, Felix Schlesinger, Jorg Drenkow, Chris Zaleski, Sonali Jha, Philippe Batut, Mark Chaisson, Thomas R Gingeras. 2013. “STAR: ultrafast universal RNA-seq aligner.” *Bioinformatics* 29: 15-21. https://doi.org/10.1093/bioinformatics/bts635

13. Brůna, Tomáš, Katharina J Hoff, Alexandre Lomsadze, Mario Stanke, Mark Borodovsky. 2021. “BRAKER2: automatic eukaryotic genome annotation with GeneMark-EP+ and AUGUSTUS supported by a protein database.” *NAR Genomics and Bioinformatics* 3: lqaa108. https://doi.org/10.1093/nargab/lqaa108

14. Gabriel, Lars, Katharina J Hoff, Tomáš Brůna, Mark Borodovsky, Mario Stanke. 2021. “TSEBRA: transcript selector for BRAKER.” *BMC Bioinformatics* 22: 1-12. https://doi.org/10.1101/2021.06.07.447316

15. Li, Heng. 2021. “New strategies to improve minimap2 alignment accuracy.” *Bioinformatics* 37: 4572-4574. https://doi.org/10.1093/bioinformatics/btab705

16. Lagesen, Karin, Peter Hallin, Einar Andreas Rødland, Hans-Henrik Stærfeldt, Torbjørn Rognes, David W Ussery. 2007. “RNAmmer: consistent and rapid annotation of ribosomal RNA genes.” *Nucleic Acids Research* 35: 3100-3108. https://doi.org/10.1093/nar/gkm160

17. Chan, Patricia P, Todd M Lowe. 2019. “tRNAscan-SE: searching for tRNA genes in genomic sequences.” *Gene Prediction: Methods and Protocols* 1-14. https://doi.org/10.1007/978-1-4939-9173-0_1

18. Nawrocki, Eric P, Sean R Eddy. 2013. “Infernal 1.1: 100-fold faster RNA homology searches.” *Bioinformatics* 29: 2933-2935. https://doi.org/10.1093/bioinformatics/btt509

19. Kalvari, Ioanna, Eric P Nawrocki, Nancy Ontiveros-Palacios, Joanna Argasinska, Kevin Lamkiewicz, Manja Marz, Sam Griffiths-Jones, Claire Toffano-Nioche, Daniel Gautheret, Zasha Weinberg. 2021. “Rfam 14: expanded coverage of metagenomic, viral and microRNA families.” *Nucleic Acids Research* 49: D192-D200. https://doi.org/10.1093/nar/gkaa1047

20. Cantalapiedra, Carlos P, Ana Hernández-Plaza, Ivica Letunic, Peer Bork, Jaime Huerta-Cepas. 2021. “eggNOG-mapper v2: functional annotation, orthology assignments, and domain prediction at the metagenomic scale.” *Molecular Biology and Evolution* 38: 5825-5829. https://doi.org/10.1093/molbev/msab293

21. Consortium, The UniProt. 2021. “UniProt: the universal protein knowledgebase in 2021.” *Nucleic Acids Research* 49: D480-D489. https://doi.org/10.1093/nar/gkaa1100

22. Buchfink, Benjamin, Klaus Reuter, Hajk-Georg Drost. 2021. “Sensitive protein alignments at tree-of-life scale using DIAMOND.” *Nature Methods* 18: 366-368. https://doi.org/10.1038/s41592-021-01101-x

23. Liao, Wen-Wei, Mobin Asri, Jana Ebler, Daniel Doerr, Marina Haukness, Glenn Hickey, Shuangjia Lu, Julian K Lucas, Jean Monlong, Haley J Abel. 2023. “A draft human pangenome reference.” *Nature* 617: 312-324. https://doi.org/10.1038/s41586-023-05896-x

24. Gao, Yang, Xiaofei Yang, Hao Chen, Xinjiang Tan, Zhaoqing Yang, Lian Deng, Baonan Wang, Shuang Kong, Songyang Li, Yuhang Cui. 2023. “A pangenome reference of 36 Chinese populations.” *Nature* 619: 112-121. https://doi.org/10.1038/s41586-023-06173-7

25. Li, Qionghou, Xin Qiao, Lanqing Li, Chao Gu, Hao Yin, Kaijie Qi, Zhihua Xie, Sheng Yang, Qifeng Zhao, Zewen Wang. 2024. “Haplotype-resolved T2T genome assemblies and pangenome graph of pear reveal diverse patterns of allele-specific expression and the genomic basis of fruit quality traits.” *Plant Communications* https://doi.org/10.1016/j.xplc.2024.101000

26. Rice, Edward S, Antton Alberdi, James Alfieri, Giridhar Athrey, Jennifer R Balacco, Philippe Bardou, Heath Blackmon, Mathieu Charles, Hans H Cheng, Olivier Fedrigo. 2023. “A pangenome graph reference of 30 chicken genomes allows genotyping of large and complex structural variants.” *BMC Biology* 21: 267. https://doi.org/10.1186/s12915-023-01758-0

27. Hickey, Glenn, Jean Monlong, Jana Ebler, Adam M Novak, Jordan M Eizenga, Yan Gao, Tobias Marschall, Heng Li, Benedict Paten. 2023. “Pangenome graph construction from genome alignments with Minigraph-Cactus.” *Nature Biotechnology* 1-11. https://doi.org/10.1038/s41587-023-01793-w

28. Garrison, Erik, Jouni Sirén, Adam M Novak, Glenn Hickey, Jordan M Eizenga, Eric T Dawson, William Jones, Shilpa Garg, Charles Markello, Michael F Lin. 2018. “Variation graph toolkit improves read mapping by representing genetic variation in the reference.” *Nature Biotechnology* 36: 875-879. https://doi.org/10.1038/nbt.4227

29. Ondov, Brian D, Todd J Treangen, Páll Melsted, Adam B Mallonee, Nicholas H Bergman, Sergey Koren, Adam M Phillippy. 2016. “Mash: fast genome and metagenome distance estimation using MinHash.” *Genome Biology* 17: 1-14. https://doi.org/10.1186/s13059-016-0997-x

30. Tian, Xiaomeng, Ran Li, Weiwei Fu, Yan Li, Xihong Wang, Ming Li, Duo Du, Qianzi Tang, Yudong Cai, Yiming Long. 2020. “Building a sequence map of the pig pan-genome from multiple de novo assemblies and Hi-C data.” *Science China Life Sciences* 63: 750-763. https://doi.org/10.1007/s11427-019-9551-7

31. Li, Zhengcao, Xiaohong Liu, Chen Wang, Zhenyang Li, Bo Jiang, Ruifeng Zhang, Lu Tong, Youping Qu, Sheng He, Haifan Chen. 2023. “The pig pangenome provides insights into the roles of coding structural variations in genetic diversity and adaptation.” *Genome Research* 33: 1833-1847. https://doi.org/10.1101/gr.277638.122

32. Du, Heng, Chenguang Diao, Yue Zhuo, Xianrui Zheng, Zhengzheng Hu, Shiyu Lu, Wenjiao Jin, Lei Zhou, Jian-Feng Liu. 2024. “Assembly of novel sequences for Chinese domestic pigs reveals new genes and regulatory variants providing new insights into their diversity.” *Genomics* 116: 110782. https://doi.org/10.1016/j.ygeno.2024.110782

33. Emms, David M, Steven Kelly. 2019. “OrthoFinder: phylogenetic orthology inference for comparative genomics.” *Genome Biology* 20: 1-14. https://doi.org/10.1186/s13059-019-1832-y

34. Kolberg, Liis, Uku Raudvere, Ivan Kuzmin, Priit Adler, Jaak Vilo, Hedi Peterson. 2023. “g: Profiler—interoperable web service for functional enrichment analysis and gene identifier mapping (2023 update).” *Nucleic Acids Research* 51: W207-W212. https://doi.org/10.1093/nar/gkad347

35. O’Connell, Kyle A, Zelaikha B Yosufzai, Ross A Campbell, Collin J Lobb, Haley T Engelken, Laura M Gorrell, Thad B Carlson, Josh J Catana, Dina Mikdadi, Vivien R Bonazzi. 2023. “Accelerating genomic workflows using NVIDIA Parabricks.” *BMC Bioinformatics* 24: 1-15. https://doi.org/10.1186/s12859-023-05292-2

36. Lin, Michael F, Ohad Rodeh, John Penn, Xiaodong Bai, Jeffrey G Reid, Olga Krasheninina, William J Salerno. 2018. “GLnexus: joint variant calling for large cohort sequencing.” *bioRxiv* 343970. https://doi.org/10.1101/343970

37. Chen, Xiaoyu, Ole Schulz-Trieglaff, Richard Shaw, Bret Barnes, Felix Schlesinger, Morten Källberg, Anthony J Cox, Semyon Kruglyak, Christopher T Saunders. 2016. “Manta: rapid detection of structural variants and indels for germline and cancer sequencing applications.” *Bioinformatics* 32: 1220-1222. https://doi.org/10.1093/bioinformatics/btv710

38. Ebler, Jana, Peter Ebert, Wayne E Clarke, Tobias Rausch, Peter A Audano, Torsten Houwaart, Yafei Mao, Jan O Korbel, Evan E Eichler, Michael C Zody. 2022. “Pangenome-based genome inference allows efficient and accurate genotyping across a wide spectrum of variant classes.” *Nature Genetics* 54: 518-525. https://doi.org/10.1038/s41588-022-01043-w

39. Chang, Christopher C, Carson C Chow, Laurent CAM Tellier, Shashaank Vattikuti, Shaun M Purcell, James J Lee. 2015. “Second-generation PLINK: rising to the challenge of larger and richer datasets.” *Gigascience* 4: s13742-13015-10047-13748. https://doi.org/10.1186/s13742-015-0047-8

40. Kumar, Sudhir, Glen Stecher, Michael Li, Christina Knyaz, Koichiro Tamura. 2018. “MEGA X: molecular evolutionary genetics analysis across computing platforms.” *Molecular Biology and Evolution* 35: 1547. https://doi.org/10.1093/molbev/msy096

41. Letunic, Ivica, Peer Bork. 2021. “Interactive Tree Of Life (iTOL) v5: an online tool for phylogenetic tree display and annotation.” *Nucleic Acids Research* 49: W293-W296. https://doi.org/10.1093/nar/gkab301

42. Alexander, David H, John Novembre, Kenneth Lange. 2009. “Fast model-based estimation of ancestry in unrelated individuals.” *Genome Research* 19: 1655-1664. https://doi.org/10.1161/01.ATV.0000137190.63214.c5

43. Dong, Shan-Shan, Wei-Ming He, Jing-Jing Ji, Chi Zhang, Yan Guo, Tie-Lin Yang. 2021. “LDBlockShow: a fast and convenient tool for visualizing linkage disequilibrium and haplotype blocks based on variant call format files.” *Briefings in Bioinformatics* 22: bbaa227. https://doi.org/10.1093/bib/bbaa227

44. McLaren, William, Laurent Gil, Sarah E Hunt, Harpreet Singh Riat, Graham RS Ritchie, Anja Thormann, Paul Flicek, Fiona Cunningham. 2016. “The ensembl variant effect predictor.” *Genome Biology* 17: 1-14. https://doi.org/10.1186/s13059-016-0974-4

45. Fick, Stephen E, Robert J Hijmans. 2017. “WorldClim 2: new 1‐km spatial resolution climate surfaces for global land areas.” *International Journal of Climatology* 37: 4302-4315. https://doi.org/10.1002/joc.5086

46. Beckmann, Michael, Tomáš Václavík, Ameur M Manceur, Lenka Šprtová, Henrik von Wehrden, Erik Welk, Anna F Cord. 2014. “gl UV: a global UV‐B radiation data set for macroecological studies.” *Methods in Ecology and Evolution* 5: 372-383. https://doi.org/10.1111/2041-210X.12168

47. Hengl, Tomislav, Jorge Mendes de Jesus, Gerard BM Heuvelink, Maria Ruiperez Gonzalez, Milan Kilibarda, Aleksandar Blagotić, Wei Shangguan, Marvin N Wright, Xiaoyuan Geng, Bernhard Bauer-Marschallinger. 2017. “SoilGrids250m: Global gridded soil information based on machine learning.” *PLoS One* 12: e0169748. https://doi.org/10.1371/journal.pone.0169748

48. Dormann, Carsten F, Jane Elith, Sven Bacher, Carsten Buchmann, Gudrun Carl, Gabriel Carré, Jaime R García Marquéz, Bernd Gruber, Bruno Lafourcade, Pedro J Leitão. 2013. “Collinearity: a review of methods to deal with it and a simulation study evaluating their performance.” *Ecography* 36: 27-46. https://doi.org/10.1111/j.1600-0587.2012.07348.x

49. Revelle, William, Maintainer William Revelle. 2015. “Package ‘psych’.” *The comprehensive R archive network* 337,

50. Caye, Kevin, Basile Jumentier, Johanna Lepeule, Olivier François. 2019. “LFMM 2: fast and accurate inference of gene-environment associations in genome-wide studies.” *Molecular Biology and Evolution* 36: 852-860. https://doi.org/10.1093/molbev/msz008

51. Oksanen, Jari. 2010. “Vegan: community ecology package.” *http://vegan. r-forge. r-project. org/*,

52. Forester, Brenna R, Jesse R Lasky, Helene H Wagner, Dean L Urban. 2018. “Comparing methods for detecting multilocus adaptation with multivariate genotype–environment associations.” *Molecular Ecology* 27: 2215-2233. https://doi.org/10.1111/mec.14584

53. Nocchi, Gabriele, Jing Wang, Long Yang, Junyi Ding, Ying Gao, Richard JA Buggs, Nian Wang. 2023. “Genomic signals of local adaptation and hybridization in Asian white birch.” *Molecular Ecology* 32: 595-612. https://doi.org/10.1111/mec.16788

54. Gain, Clément, Olivier François. 2021. “LEA 3: Factor models in population genetics and ecological genomics with R.” *Molecular Ecology Resources* 21: 2738-2748. https://doi.org/10.1111/1755-0998.13366

55. Wang, L, A Wang, L Wang, K Li, G Yang, R He, L Qian, N Xu, R Huang, Z Peng. 2011. “Animal genetic resources in China: pigs.” *China Agric Ture Press* 5: 25-29.

56. Čandek-Potokar, Marjeta, Rosa Nieto. 2019. European local pig breeds-diversity and performance: A study of project treasure. *BoD–Books on Demand* https://doi.org/10.5772/intechopen.83749

57. Hu, Zhi-Liang, Carissa A Park, James M Reecy. 2022. “Bringing the Animal QTLdb and CorrDB into the future: Meeting new challenges and providing updated services.” *Nucleic Acids Research* 50: D956-D961. https://doi.org/10.1093/nar/gkab1116

58. Zeng, Haonan, Wenjing Zhang, Qing Lin, Yahui Gao, Jinyan Teng, Zhiting Xu, Xiaodian Cai, Zhanming Zhong, Jun Wu, Yuqiang Liu. 2024. “PigBiobank: a valuable resource for understanding genetic and biological mechanisms of diverse complex traits in pigs.” *Nucleic Acids Research* 52: D980-D989. https://doi.org/10.1093/nar/gkad1080

59. Huang, Yizhong, Lisheng Zhou, Junjie Zhang, Xianxian Liu, Yifeng Zhang, Liping Cai, Wanchang Zhang, Leilei Cui, Jie Yang, Jiuxiu Ji. 2020. “A large-scale comparison of meat quality and intramuscular fatty acid composition among three Chinese indigenous pig breeds.” *Meat Science* 168: 108182. https://doi.org/10.1016/j.meatsci.2020.108182

60. Sun, Yunmei, Rui Cai, Yingqian Wang, Rui Zhao, Jin Qin, Weijun Pang. 2020. “A newly identified LncRNA LncIMF4 controls adipogenesis of porcine intramuscular preadipocyte through attenuating autophagy to inhibit lipolysis.” *Animals* 10: 926. https://doi.org/10.3390/ani10060926

**Supplementary figures**

Figure S1. Pig genome assembly and pangenome analysis. (A) Genome collinearity analysis. (B) Statistical results of repetitive sequences in the 27 pig genomes. (C) Benchmarking universal single-copy orthologs (BUSCO) evaluation results for the 27 pig genomes. (D) Sequence added to the graph by each sample, with samples ordered by how much sequence they contribute to the assembly generated by Minigraph-Cactus. **(E)** Comparative statistics of pig pangenome sequences derived from different methods.


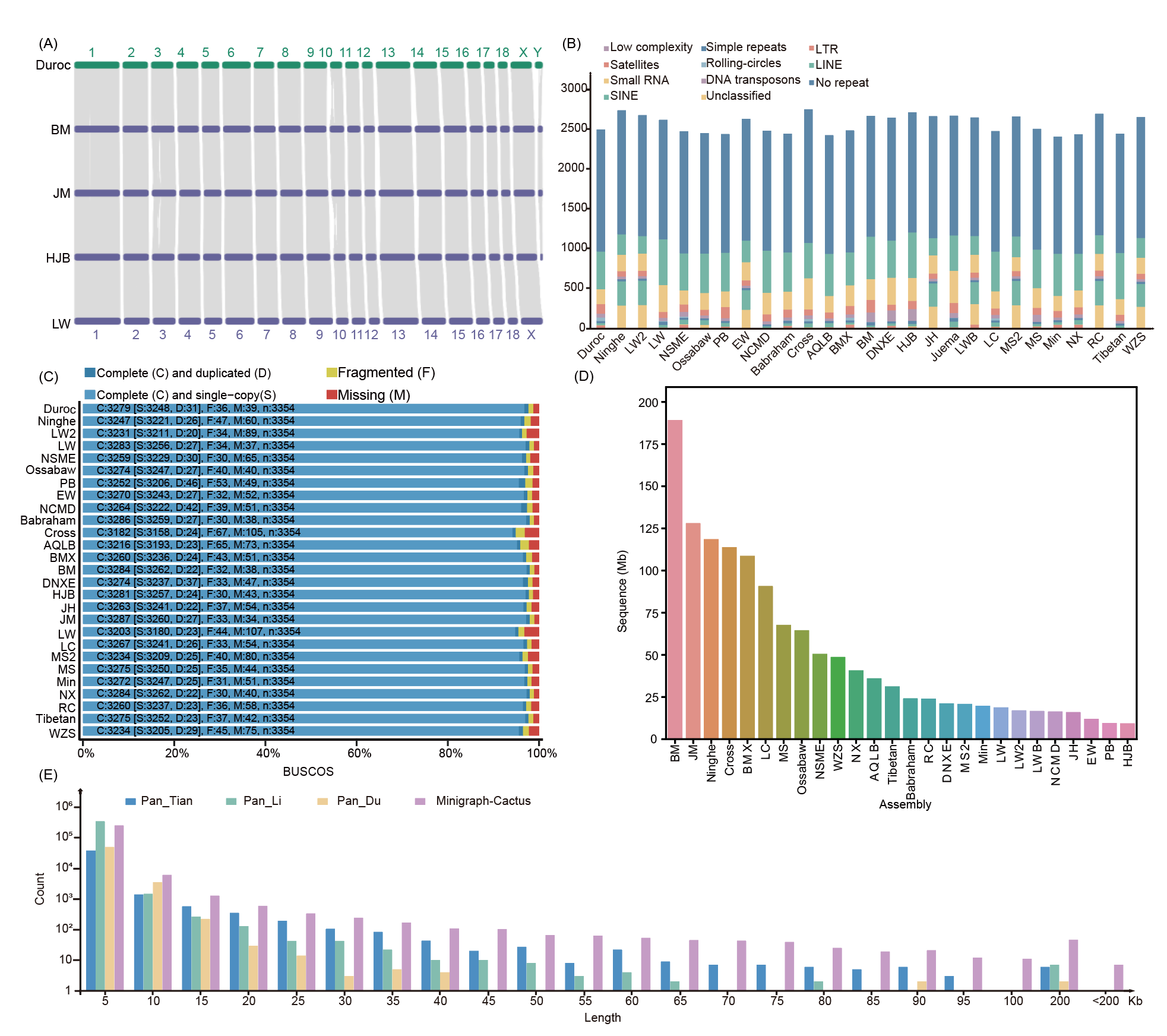


Figure S2. Gene enrichment analysis of pig pangenome genes. (A) Gene Ontology (GO) enrichment analysis of pangenome gene families. (B) Kyoto Encyclopedia of Genes and Genomes (KEGG) enrichment analysis of pangenome gene families. **(C)** GO enrichment analysis of private gene families. **(D)** Kyoto Encyclopedia of Genes and Genomes (KEGG) and GO enrichment analysis of core gene families.


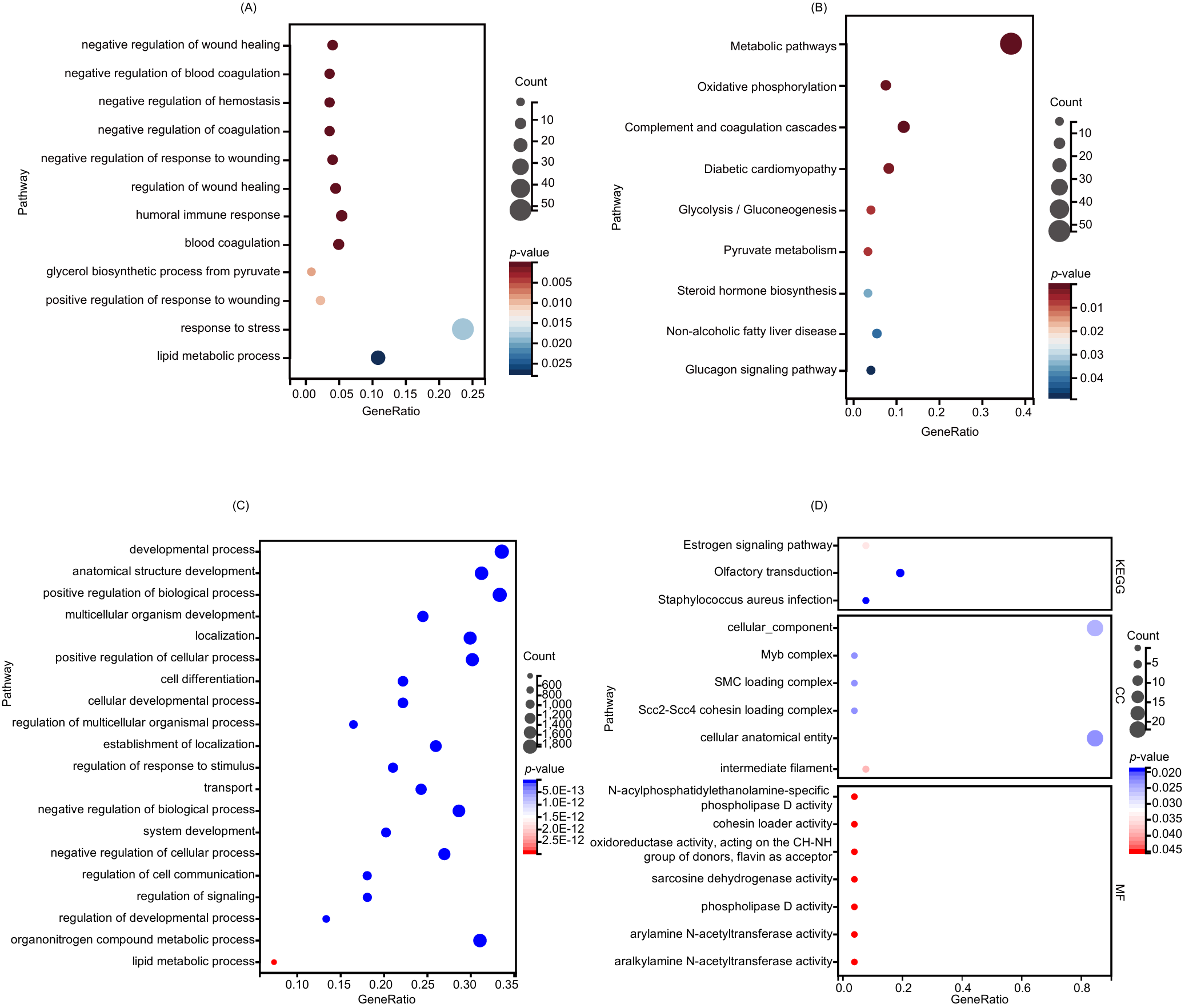


**Figure S3. Results of structural variations (SVs) analysis**. **(A)** Length statistics of insertions (INS) and deletions (DEL) within SV sets detected by Pangenie and Manta. **(B)** Ensembl Variant Effect Predictor (VEP) annotations for SVs detected by Pangenie. **(C)** VEP annotations for SVs detected by Manta. **(D)** Admixture analysis of 598 pig accessions (K = 2, K = 4) with SNPs, Indels, and SVs detected by Pangenie and Manta.


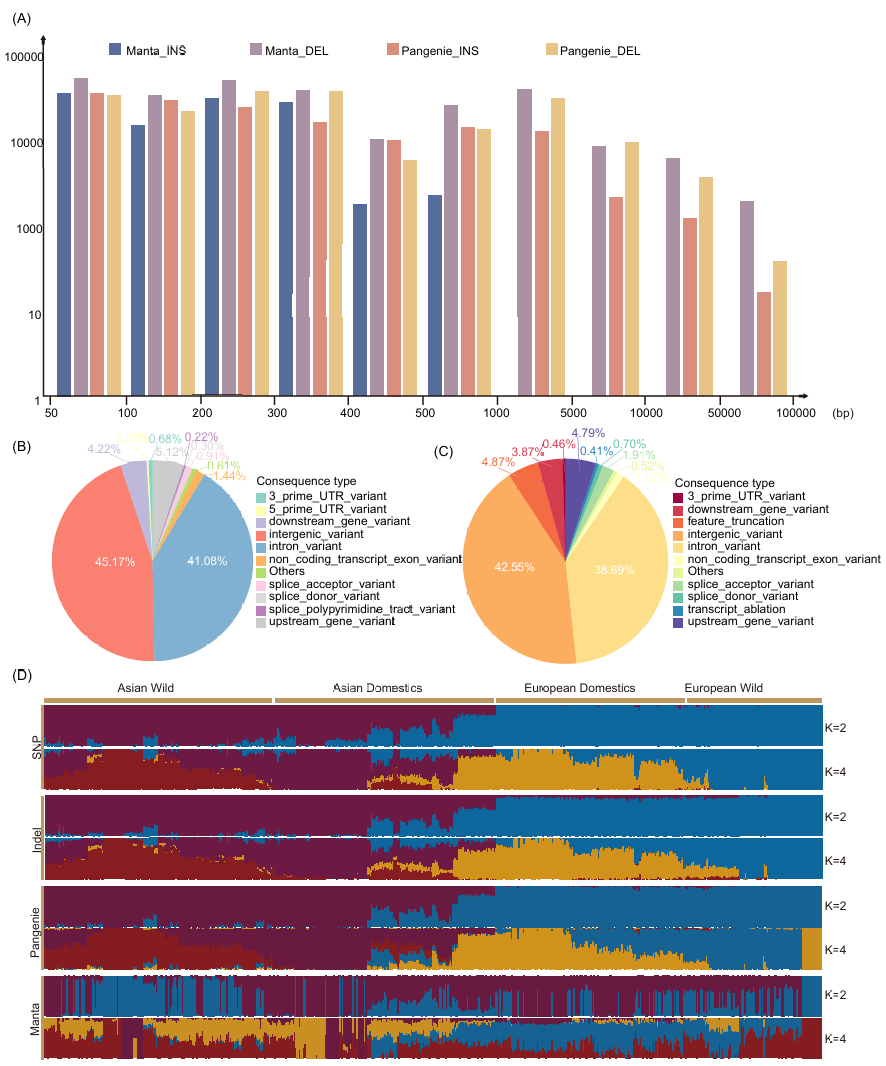


**Figure S4. Analysis of the population structure of Pangenie's SVs. (A)** Neighbor-joining (NJ) tree based on Pangenie analysis of 598 pigs. **(B)** Principal component analysis (PCA) plot of 598 pigs derived from Pangenie results. **(C)** Admixture analysis of 598 pigs showing population genetic structure based on Pangenie results. The length of each colored segment represents the proportion of an individual’s genome originating from K = 2 to 10 ancestral populations. **(D)** Cross-validation error for K values ranging from 2 to 10.


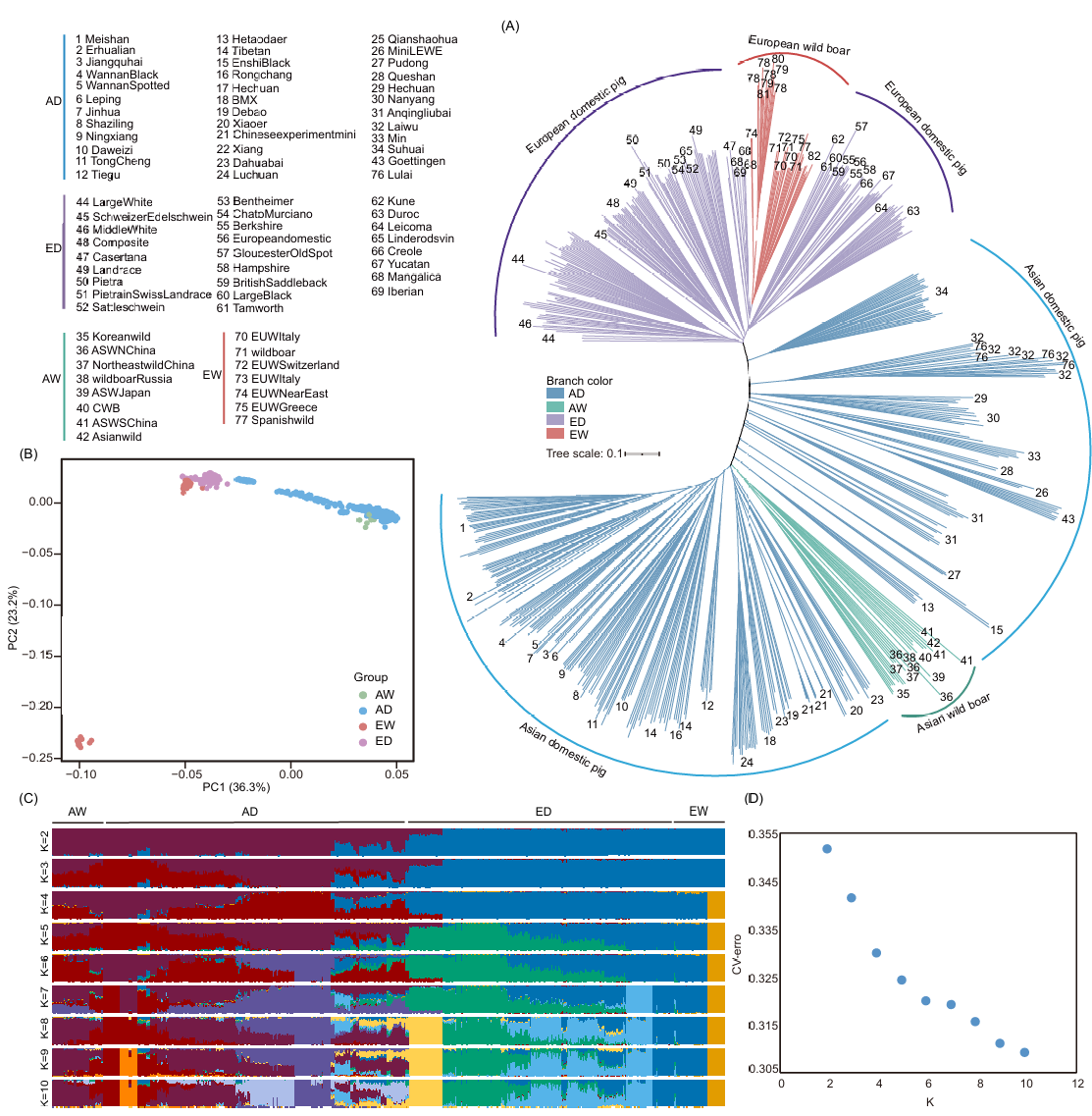


**Figure S5.** **Analysis of the population structure of Manta's** **SVs.** **(A)** NJ tree based on Manta analysis of 598 pigs. **(B)** PCA plot of 598 pigs derived from Manta results. **(C)** Admixture analysis of 598 pigs showing population genetic structure based on Manta results. The length of each colored segment represents the proportion of an individual’s genome originating from K = 2 to 10 ancestral populations. **(D)** Cross-validation error for K values ranging from 2 to 10.


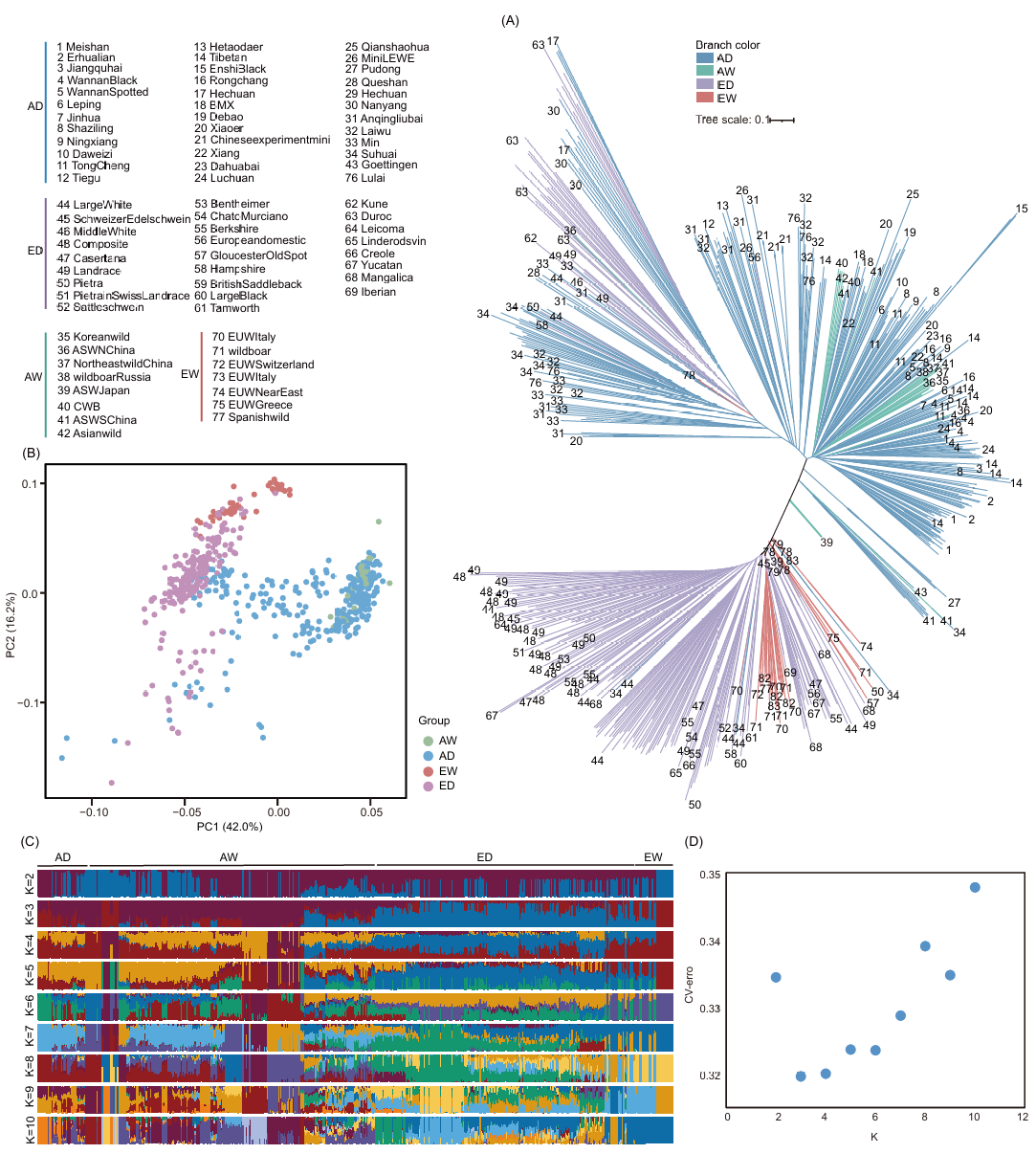


**Figure S6.** **Analysis of the population structure of single-nucleotide polymorphisms (SNPs). (A)** NJ tree based on SNPs analysis of 598 pigs and 5 outgroups. **(B)** PCA plot of 598 pigs derived from SNPs results. **(C)** Admixture analysis of 598 pigs showing population genetic structure based on SNPs results. The length of each colored segment represents the proportion of an individual’s genome originating from K = 2 to 10 ancestral populations. **(D)** Cross-validation error for K values ranging from 2 to 10.


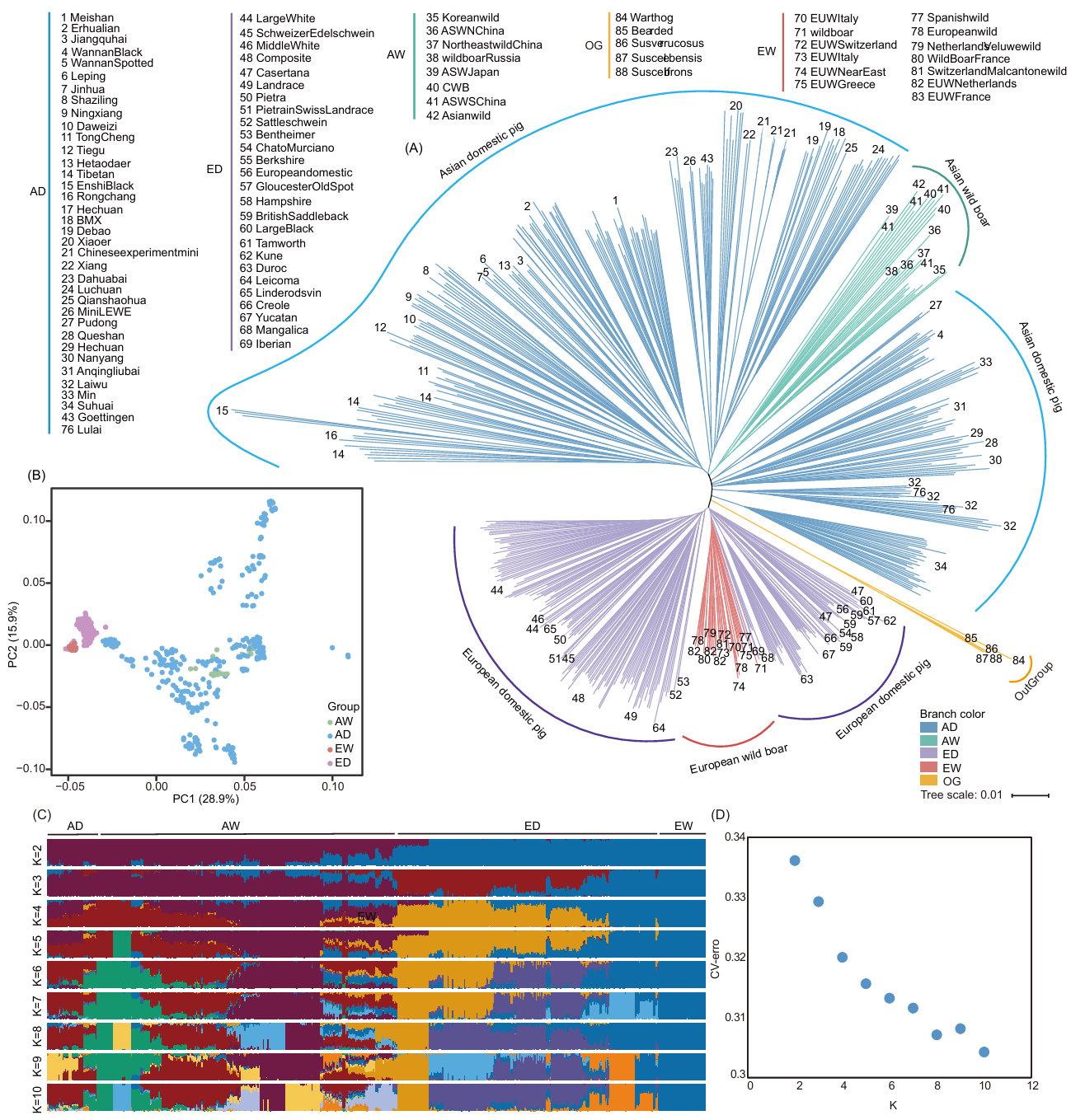


**Figure S7.** **Analysis of the population structure of insertions / deletions (Indels).** **(A)** NJ tree based on Indels analysis of 598 pigs and 5 outgroups. **(B)** PCA plot of 598 pigs derived from Indels results. **(C)** Admixture analysis of 598 pigs showing population genetic structure based on Indels results. The length of each colored segment represents the proportion of an individual’s genome originating from K = 2 to 10 ancestral populations. **(D)** Cross-validation error for K values ranging from 2 to 10.


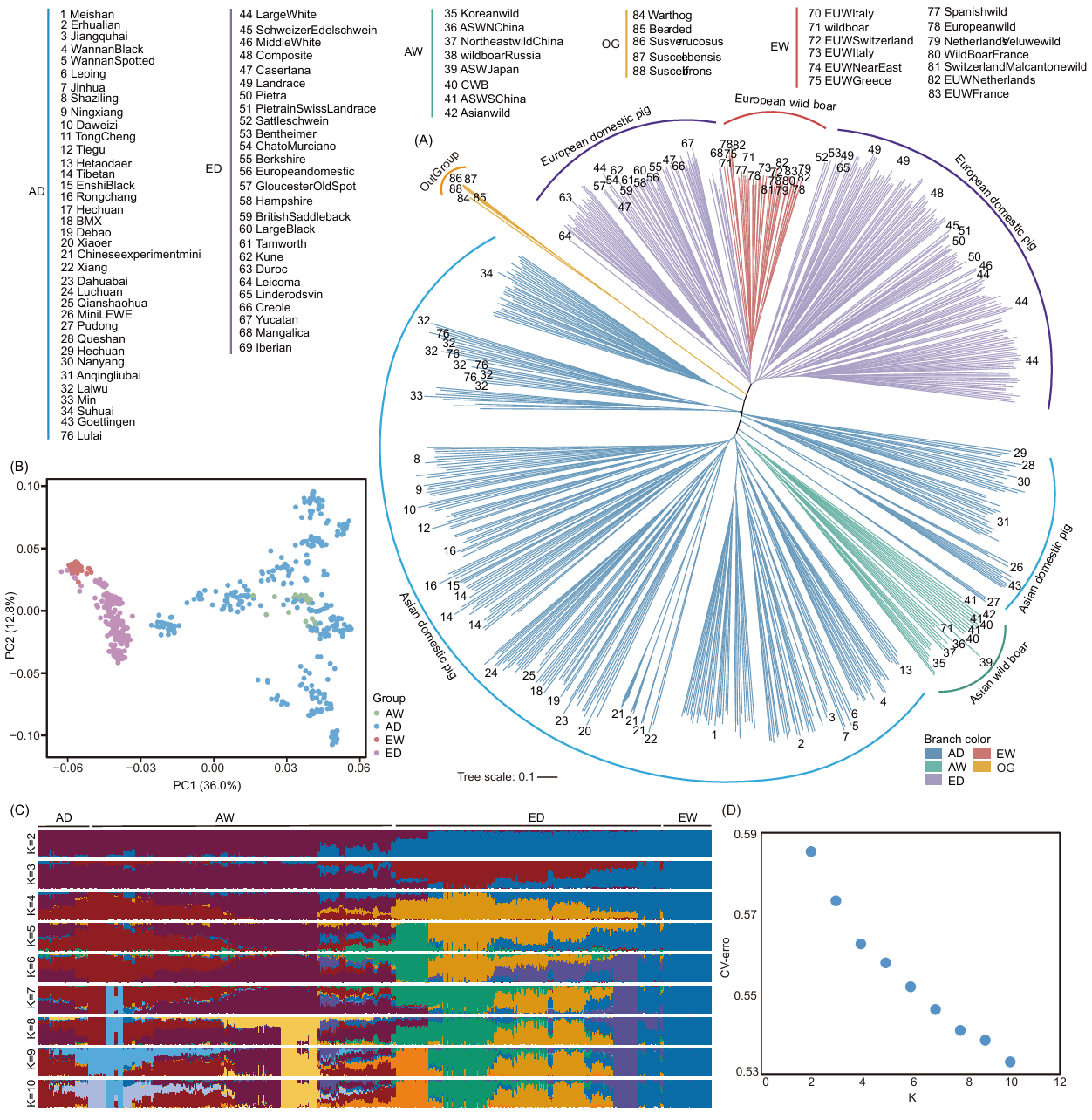


**Figure S8. Correlation analysis of 16 climate variables with “|r^2^| < 0.7”.**


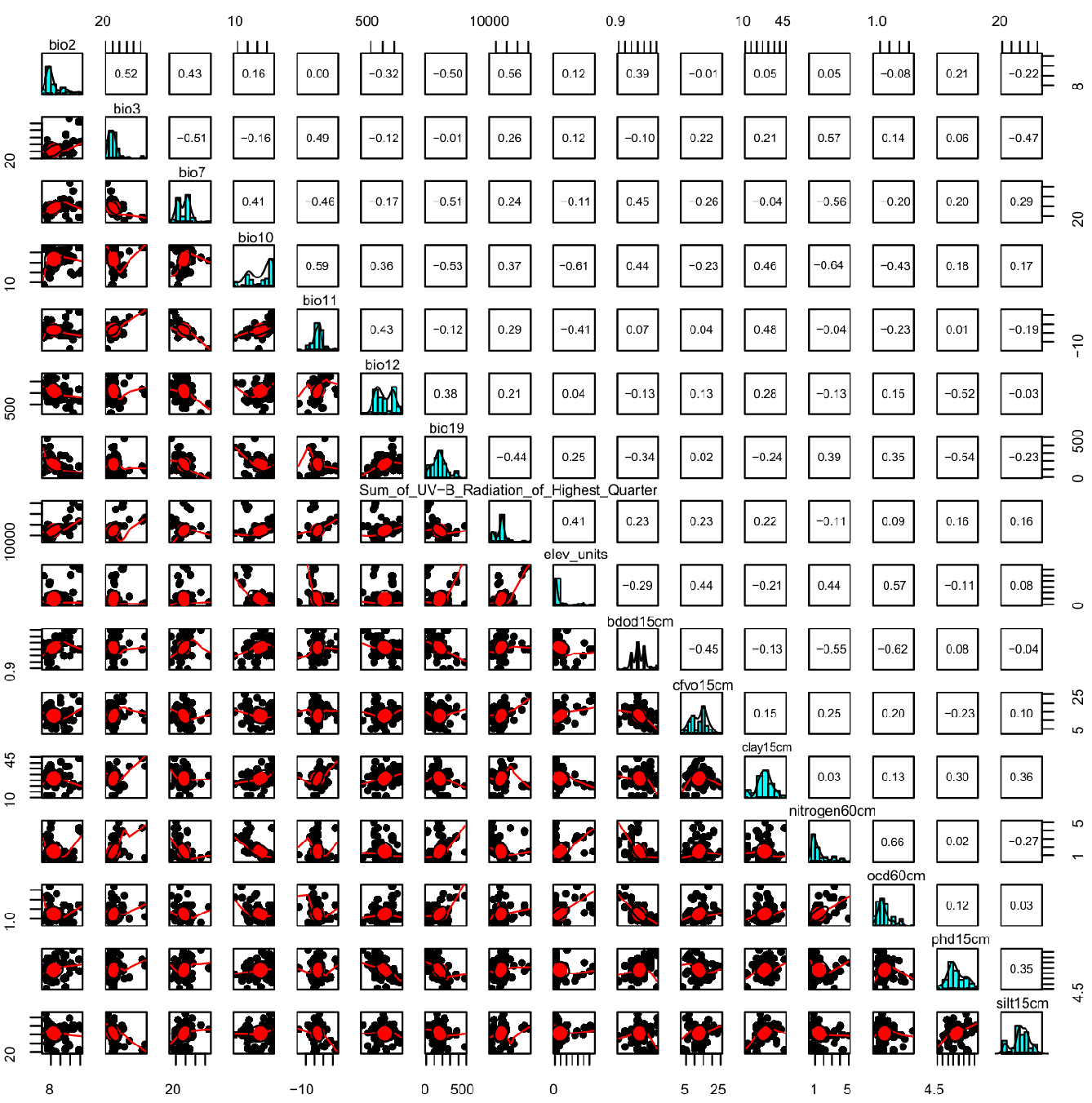


**Figure S9.** **Gene Ontology (GO) enrichment of “core adaptive genes”.**


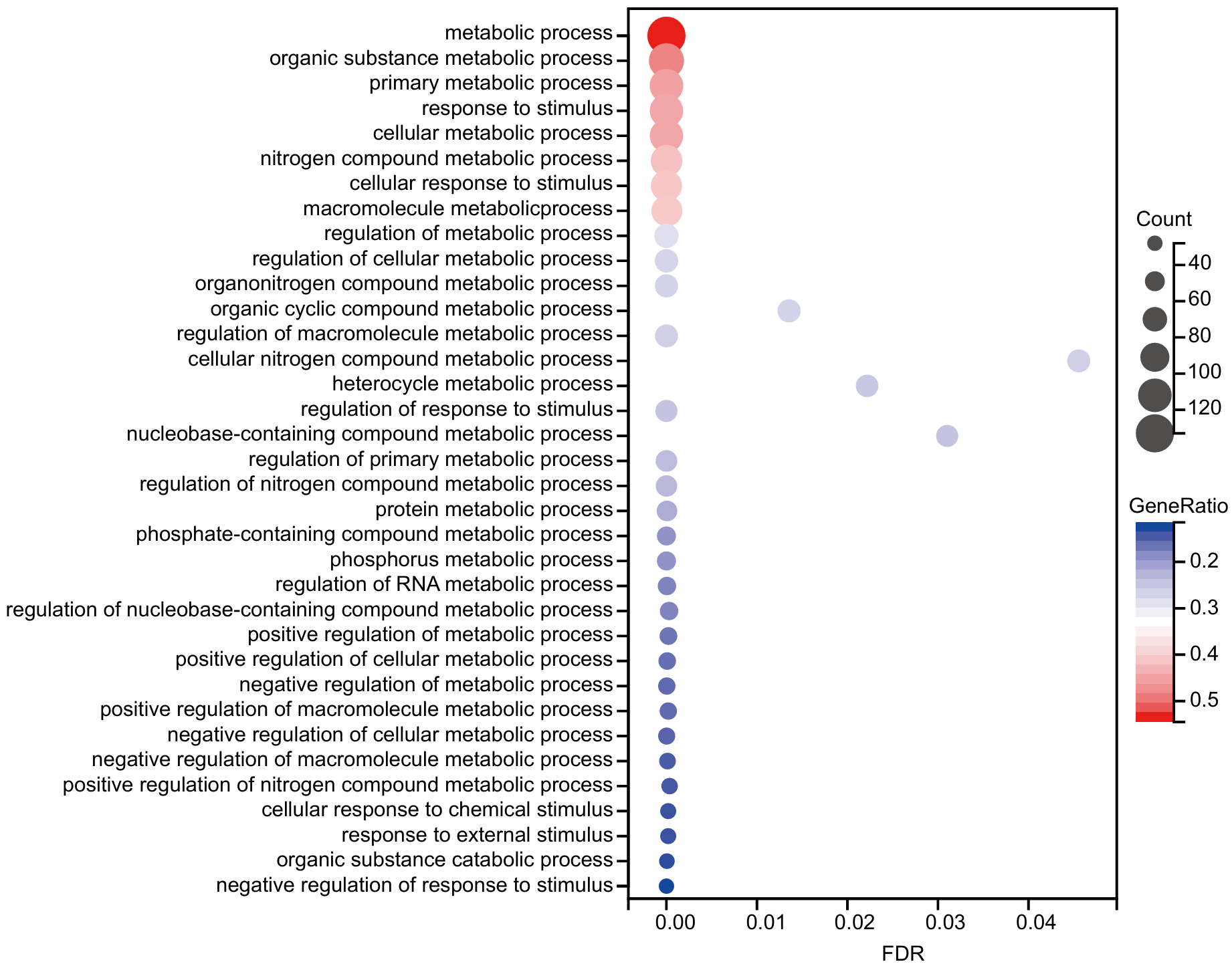


**Figure S10.** **Pig genome-environment association analyses.** **(A)** Stacked bar plot of the percentage of associated SNPs, Indels, and SVs in eco1-eco16. **(B)** Pig SVs data for RDA axes 1 and 2. **(C)** Plots of -log_10_(*p*) from genome-wide association mapping between SVs and environmental variables. top: Eco4; middle: Eco5; bottom: Eco6. Significant sites are marked in green. Significant sites are highlighted in green. Student’s t-test was used to determine significance * *p* < 0.05. ** *p* < 0.01.


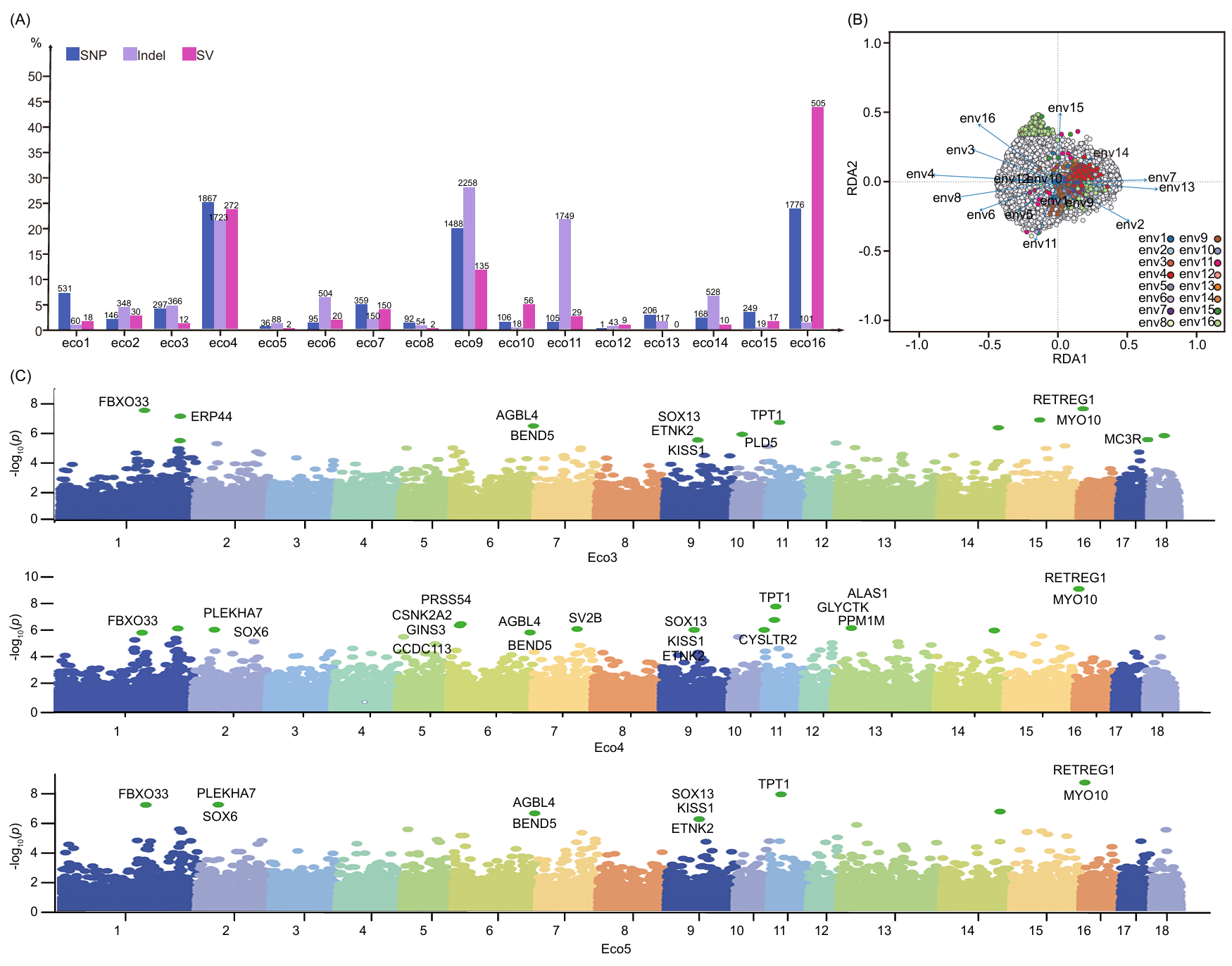


**Figure S11.** **The latent factor mixed models (LFMM) analysis of Eco1-Eco4.** **(A)** The LFMM analysis of Eco1 with SVs, SNPs and Indels. **(B)** The LFMM analysis of Eco2 with SVs, SNPs and Indels. **(C)** The LFMM analysis of Eco3 with SVs, SNPs and Indels. **(D)** The LFMM analysis of Eco4 with SVs, SNPs and Indels.


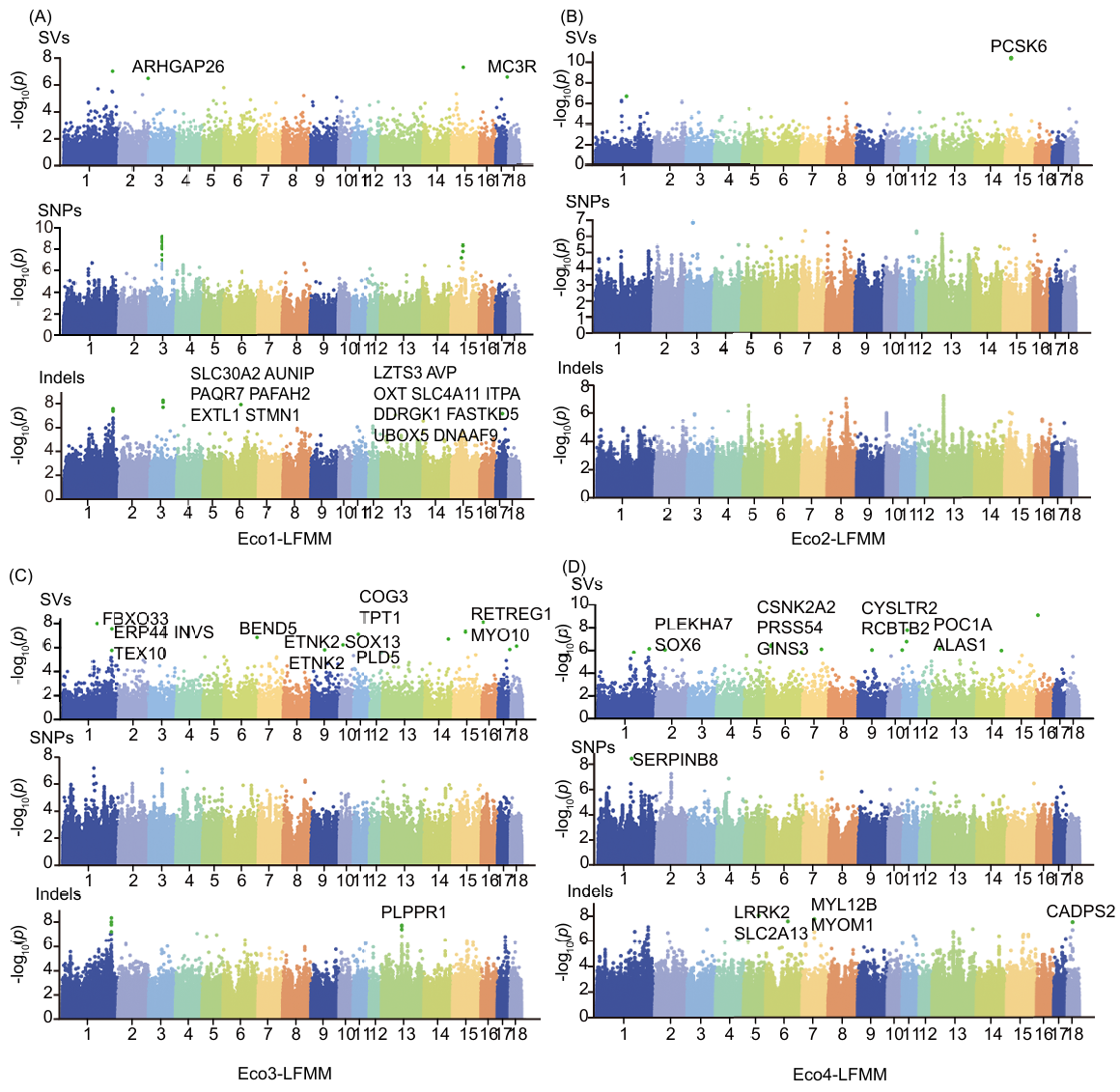


**Figure S12.** **The LFMM analysis of Eco5-Eco8. (A)** The LFMM analysis of Eco5 with SVs, SNPs and Indels. **(B)** The LFMM analysis of Eco6 with SVs, SNPs and Indels. **(C)** The LFMM analysis of Eco7 with SVs, SNPs and Indels. **(D)** The LFMM analysis of Eco8 with SVs, SNPs and Indels.


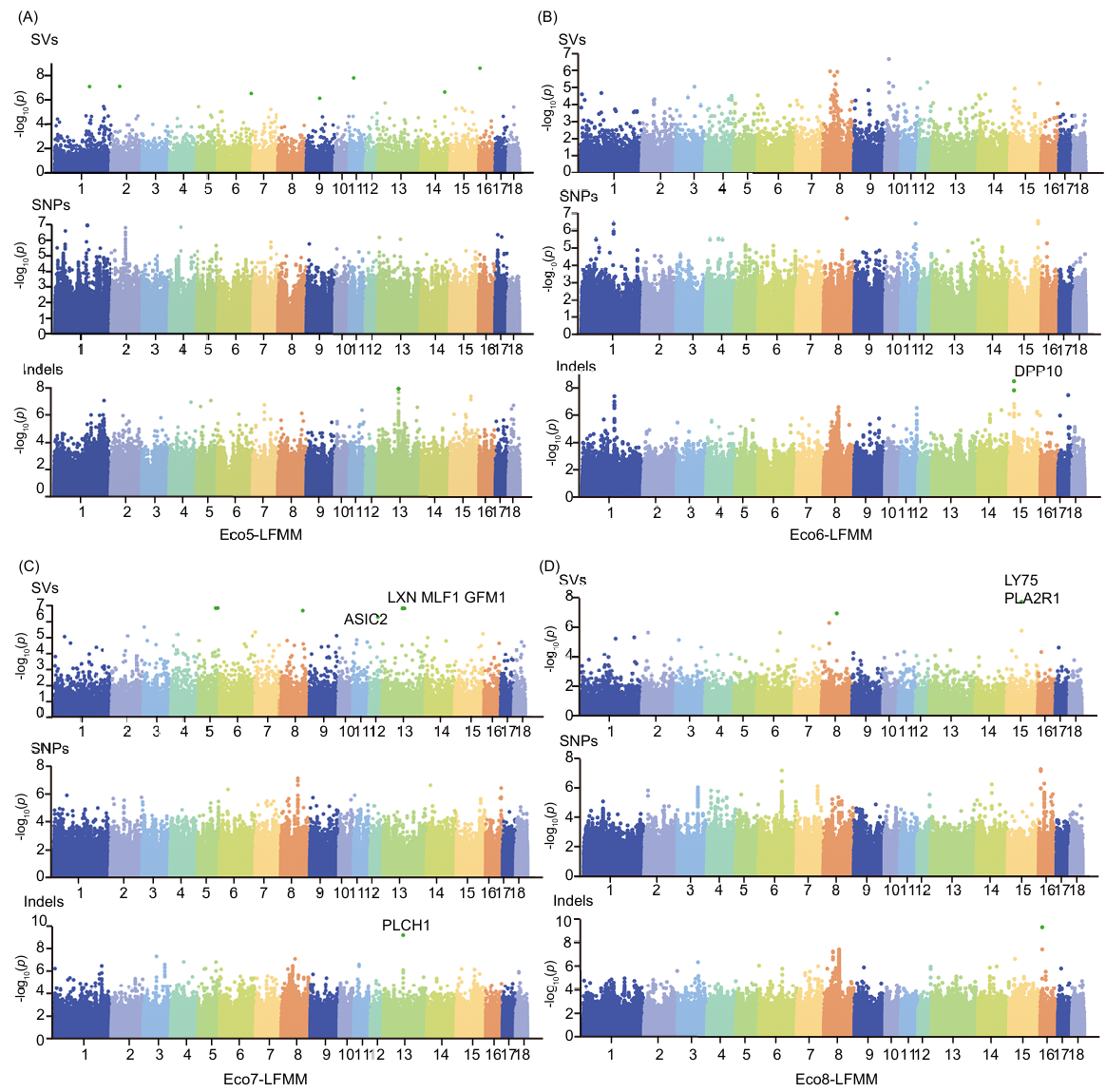


**Figure S13.** **The LFMM analysis of Eco9-Eco12. (A)** The LFMM analysis of Eco9 with SVs, SNPs and Indels. **(B)** The LFMM analysis of Eco10 with SVs, SNPs and Indels. **(C)** The LFMM analysis of Eco11 with SVs, SNPs and Indels. **(D)** The LFMM analysis of Eco12 with SVs, SNPs and Indels.


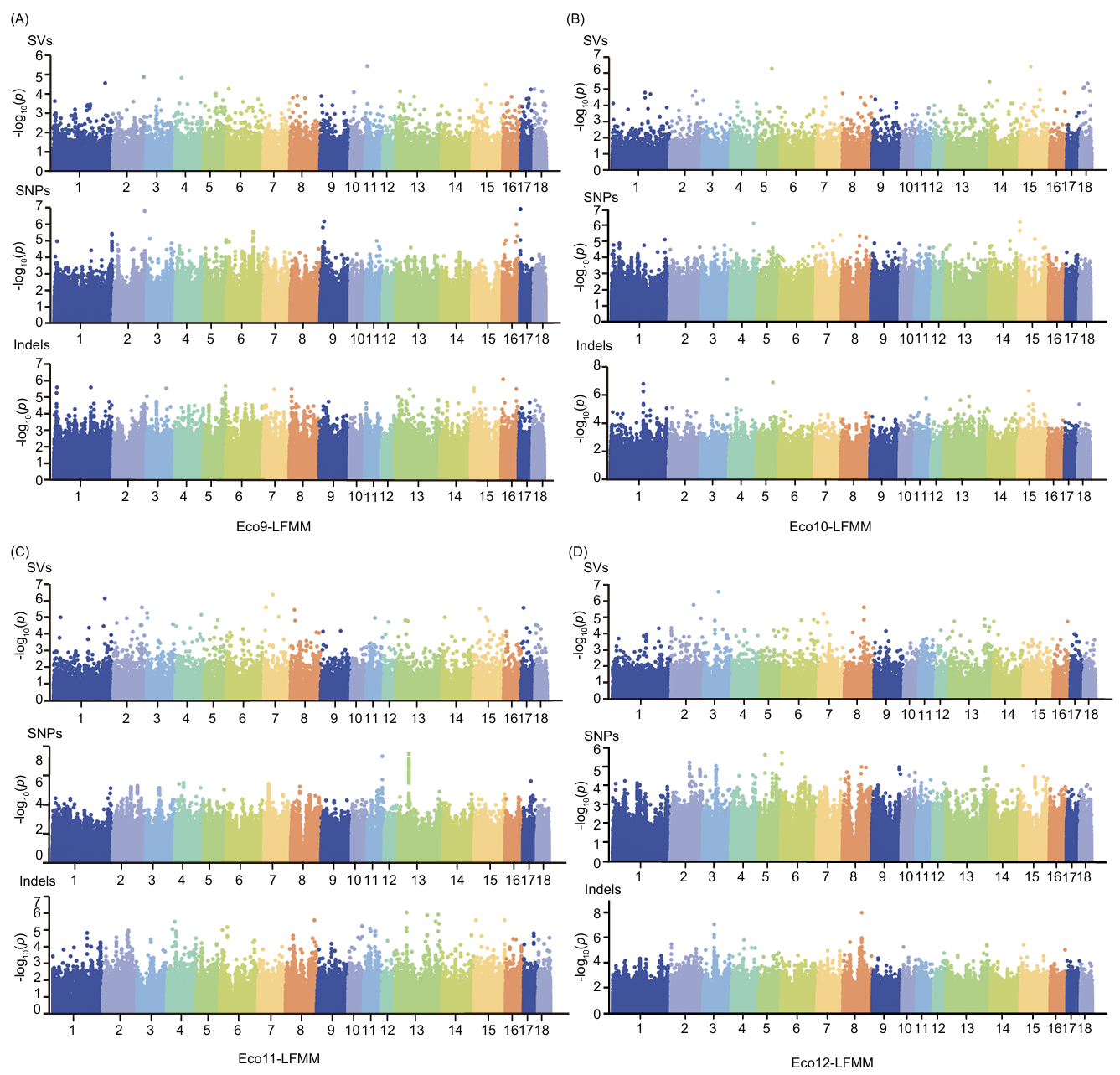


**Figure S14.** **The LFMM analysis of Eco13-Eco16.** **(A)** The LFMM analysis of Eco13 with SVs, SNPs and Indels. **(B)** The LFMM analysis of Eco14 with SVs, SNPs and Indels. **(C)** The LFMM analysis of Eco15 with SVs, SNPs and Indels. **(D)** The LFMM analysis of Eco16 with SVs, SNPs and Indels.


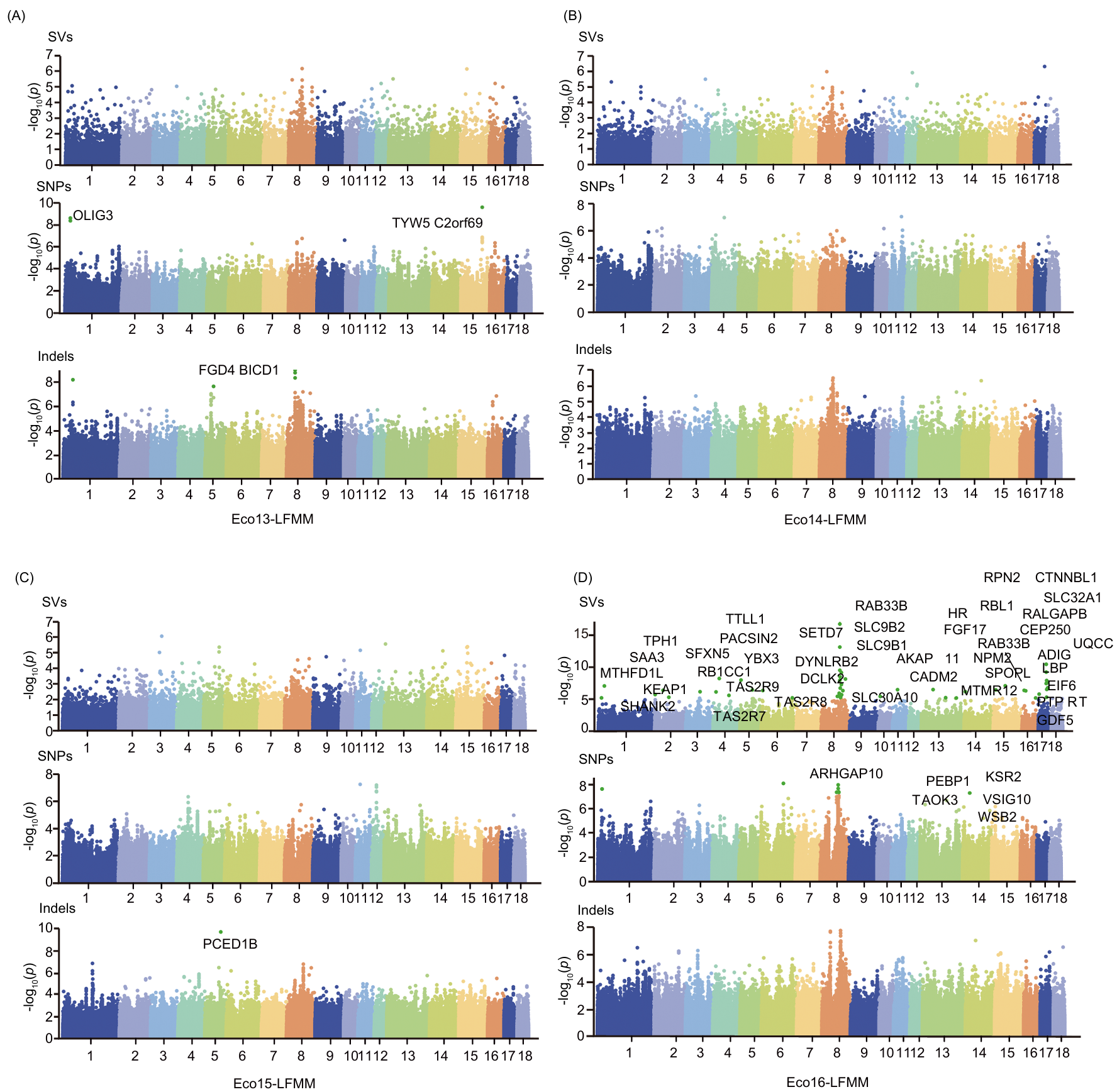


**Figure S15. Enrichment analyses of adaptive genes. (A)** Gene Ontology (GO) enrichment analysis of candidate genes identified through LFMM analysis. **(B)** Phenome-wide association (PheWAS) analysis of the relationship between candidate genes and adaptation traits


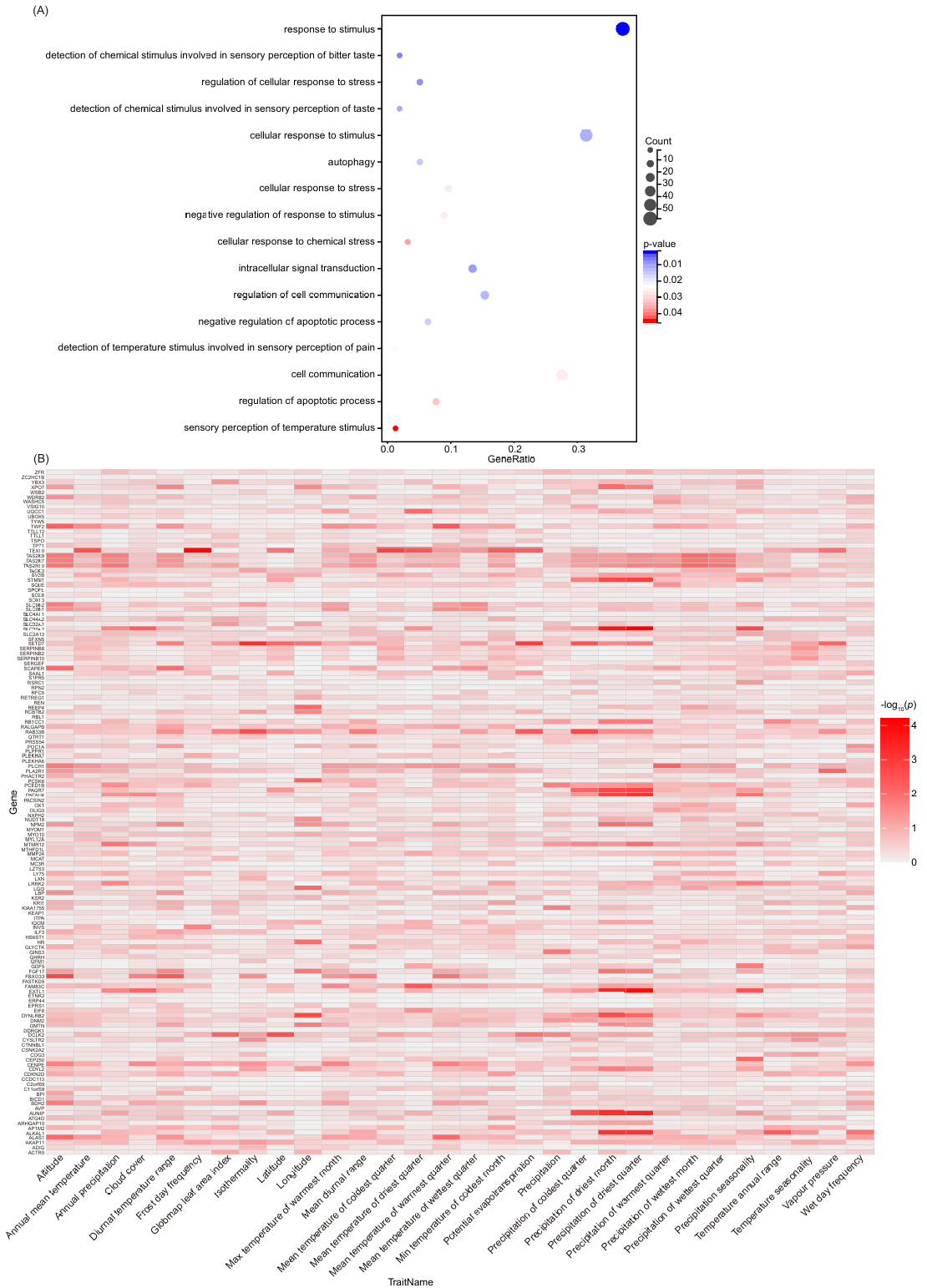


**Figure S16.** **Selection signature analysis of pig body size.** **(A)** Manhattan plots showing high and low positive selected regions by Fst (normal/mini-group) with sliding window = 50 kb, step = 25 kb, based on SVs. **(B)** Manhattan plots showing high and low positive selected regions by nucleotide diversity (normal/mini-group) with sliding window = 50 kb, step = 25 kb, based on SVs. **(C)** Gene Ontology (GO) enrichment analysis of candidate genes related to SVs identified through Fst analysis in the normal/mini groups. **(D)** Gene Ontology (GO) enrichment analysis of candidate genes related to SVs identified through π analysis in the normal/mini groups. **(E)** The PheWAS analysis of *OPA1* with production trait.


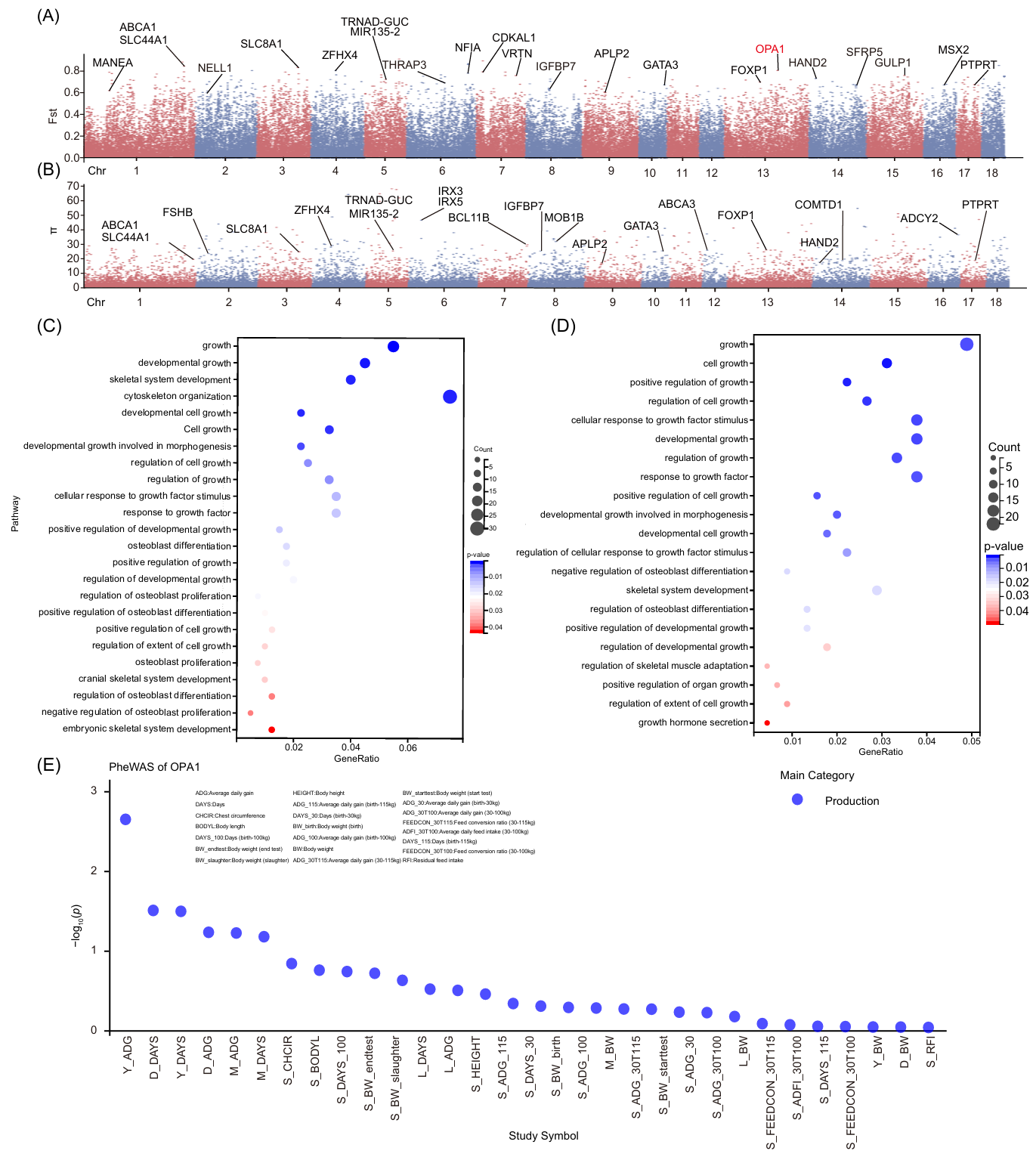


**Figure S17.** **Phenome-wide association (PheWAS) analysis of genes enriched in growth-related pathways with production traits.**


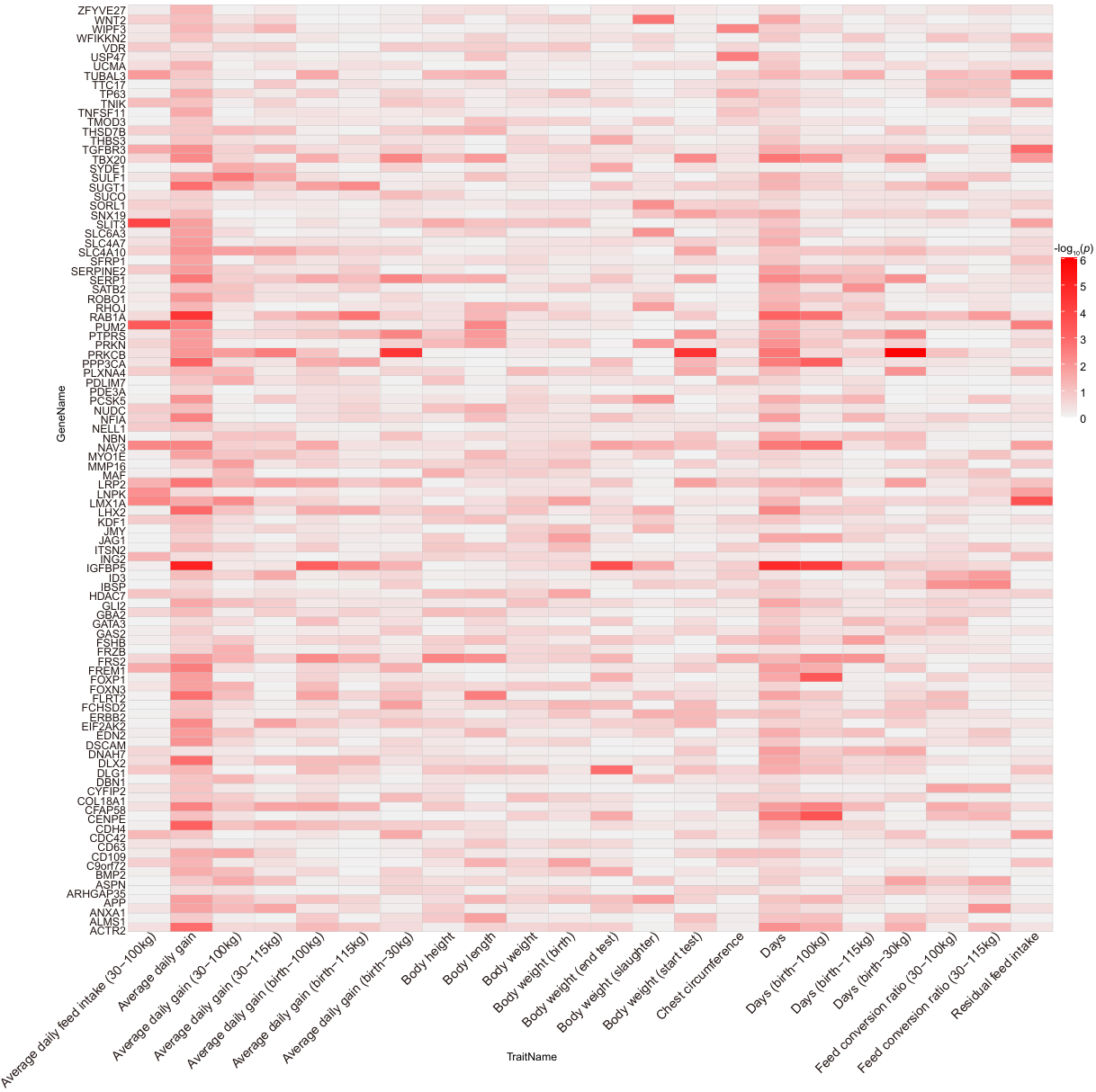


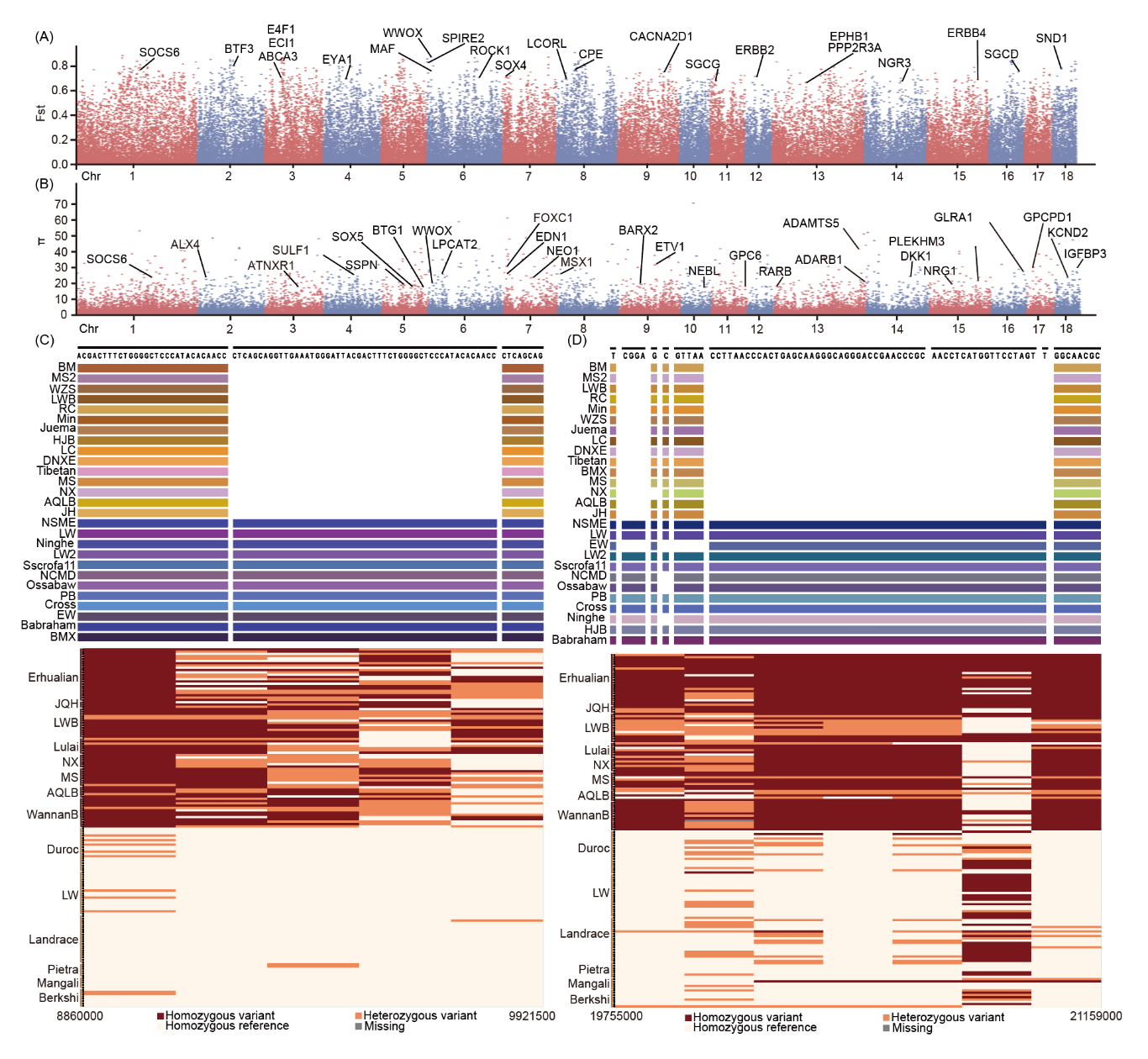
**Figure S18. Selection signature analysis of intramuscular fat (IMF).** **(A)** Manhattan plots showing high and low positive selected regions by Fst (high/low IMF group) with sliding window = 50 kb, step = 25 kb, based on SVs. **(B)** Manhattan plots showing high and low positive selected regions by nucleotide diversity (high/low IMF group) with sliding window = 50 kb, step = 25 kb, based on SVs. **(C)** Pangenomic and haplotype analysis of *WWOX*. **(D)** Pangenomic and haplotype analysis of *SND1*.

**Figure S19. Analysis of candidate genes associated with IMF.** **(A)** Gene Ontology (GO) enrichment analysis of candidate genes related to SVs identified through Fst analysis (high/low IMF groups). **(B)** Gene Ontology (GO) enrichment analysis of candidate genes related to SVs identified through π analysis (high/low IMF groups). **(C)** The PheWAS analysis of WWOX with production trait. **(D)** The PheWAS analysis of SND1 with meat and carcass traits. **(E)** The PheWAS analysis of BTF3 with meat and carcass traits.


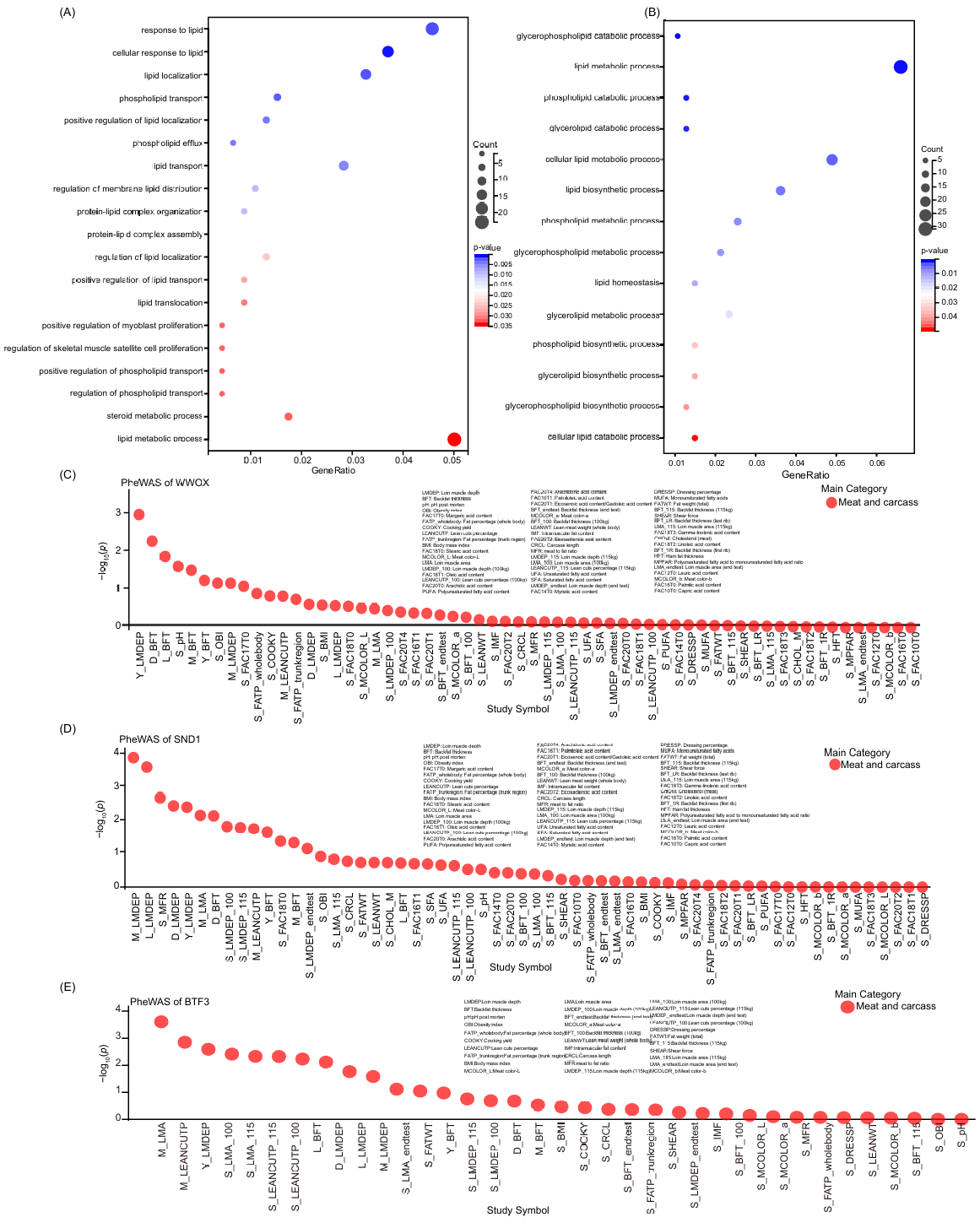


**Figure S20.** **PheWAS analysis of genes enriched in** **lipid metabolic process based on meat and carcass traits.**


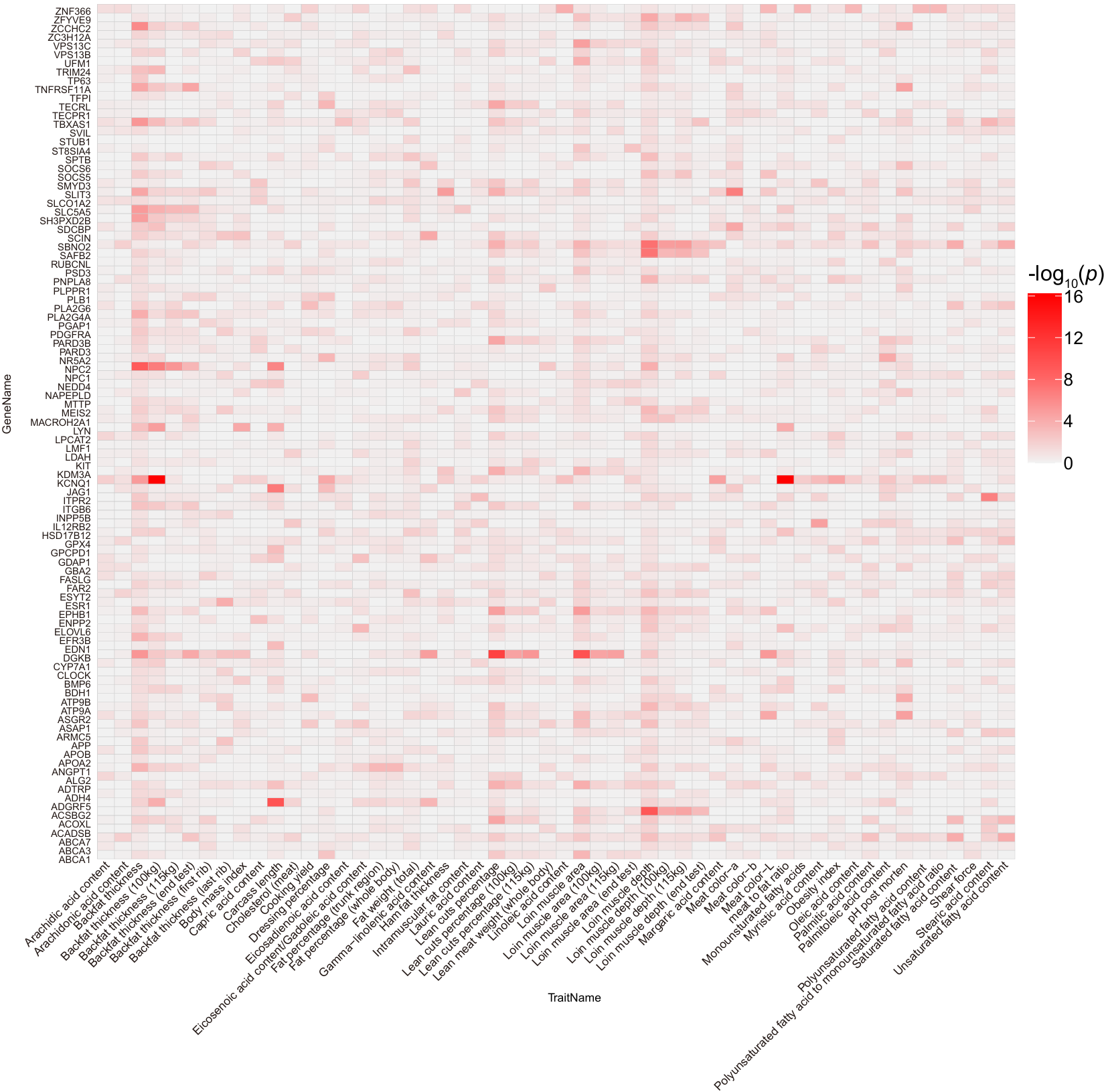


Figure S21**.** **Functional analysis of IMF-associated candidate genes.** **(A)** Real-time Quantitative PCR was used to measure the relative mRNA expression of candidate genes in Laiwu Black and Large White pigs (n = 5). **(B)** Western blot used to detect the protein expression of Ki67, CyclinD, and CyclinE after overexpressing *BTF3*, and performs grayscale analysis performed using Image J. **(C)** CCK8 assay detects the proliferation of porcine intramuscular adipose primary cells after overexpressing *BTF3*. **(D)** RT-qPCR measures the mRNA expression of *BTF3*, Ki67, CDK4 and CDK6 after interfering with *BTF3*. **(E)** Western blot detects the protein expression of Ki67, CyclinD, and CyclinE after interfering with *BTF3*, and performs grayscale analysis through Image J. **(F)** CCK8 detects the proliferation of primary pig intramuscular fat cells after interfering with *BTF3*. **(G, H)** EdU staining assay detects the proliferation of primary pig intramuscular fat cells after transfection with *BTF3*. Cells in the S phase are stained with EdU (green). Nuclei were stained with Hoechst (blue) and counted using Image J. **(I)** Western blot used to detect the protein expression of differentiation-related genes after overexpression of *BTF3*. **(J)** RT-qPCR measures the mRNA expression of differentiation-related genes after interfering with *BTF3*. **(K)** Western blot used to detect the protein expression of differentiation-related genes after interfering with *BTF3*. **(L)** Oil Red O staining detects lipids after interference with the *BTF3* formation of drops. Student’s t-test was used to determine significance * *p* < 0.05. ** *p* <0.01.


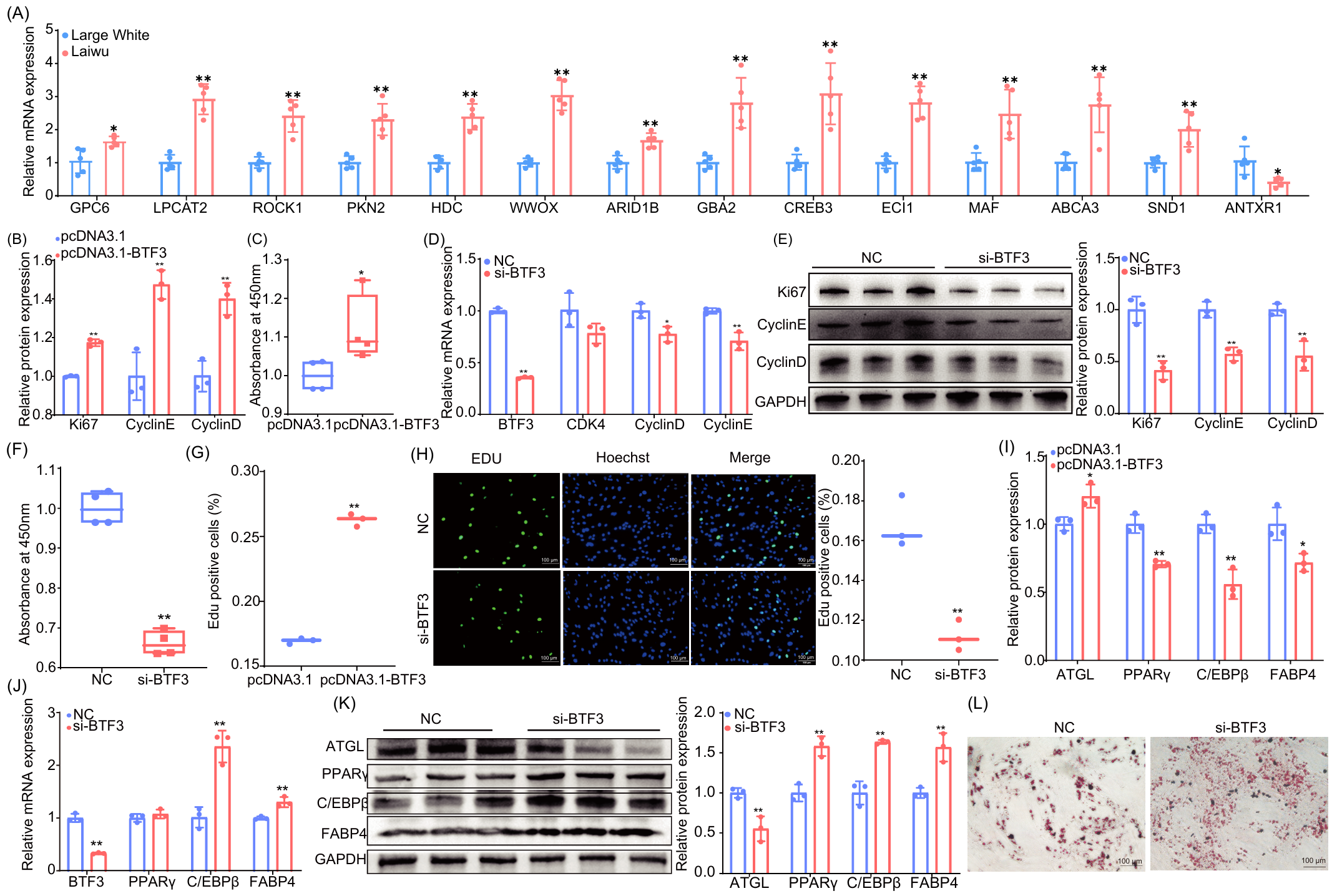

Supplement: Supplementary file 1 — Figure S1. Pig genome assembly and pangenome analyses. Figure S2. Gene enrichment analysis of pig pangenome genes. Figure S3. Results of structural variation (SV) analysis. Figure S4. Analysis of the population structure of Pangenie's SVs. Figure S5. Analysis of the population structure of Manta's SVs. Figure S6. Analysis of the population structure of single‐nucleotide polymorphisms (SNPs). Figure S7. Analysis of the population structure of insertions/deletions (Indels). Figure S8. Correlation analysis of 16 climate variables with “|r2| < 0.7”. Figure S9. Gene Ontology (GO) enrichment of “core adaptive genes”. Figure S10. Pig genome–environment association analyses. Figure S11. Latent factor mixed model (LFMM) analysis of Eco1‐Eco4. Figure S12. LFMM analysis of Eco5‐Eco8. Figure S13. LFMM analysis of Eco9‐Eco12. Figure S14. LFMM analysis of Eco13‐Eco16. Figure S15. Enrichment analyses of adaptive genes. Figure S16. Selection signature analysis of pig body size. Figure S17. Phenome‐wide association (PheWAS) analysis of genes enriched in growth‐related pathways with production traits. Figure S18. Selection signature analysis of intramuscular fat (IMF). Figure S19. Analysis of candidate genes associated with IMF. Figure S20. PheWAS analysis of genes enriched in lipid metabolic process based on meat and carcass traits. Figure S21. Functional analysis of IMF‐associated candidate genes. [file IMT2-3-e257-s001.docx]
